# Active fungal GH115 α-glucuronidase produced in *Arabidopsis thaliana* affects only the UX1-reactive glucuronate decorations on native glucuronoxylans

**DOI:** 10.1186/s12896-015-0154-8

**Published:** 2015-06-18

**Authors:** Sun-Li Chong, Marta Derba-Maceluch, Sanna Koutaniemi, Leonardo D Gómez, Simon J McQueen-Mason, Maija Tenkanen, Ewa J Mellerowicz

**Affiliations:** Department of Food and Environmental Sciences, Faculty of Agriculture and Forestry, University of Helsinki, P.O. Box 27, Helsinki, 00014 Finland; Umeå Plant Science Centre, Department of Forest Genetics and Plant Physiology, Swedish University of Agricultural Sciences, Umeå, 901-83 Sweden; Center for Novel Agricultural Products Department of Biology, University of York, York, YO10 5DD UK

**Keywords:** Xylan acetylation, Alpha-glucuronidase, 4-*O*-methylglucuronic acid, Glucuronoxylan, Xylan degradation, Feedstocks for biofuels, Secondary cell walls

## Abstract

**Background:**

Expressing microbial polysaccharide-modifying enzymes in plants is an attractive approach to custom tailor plant lignocellulose and to study the importance of wall structures to plant development. Expression of α-glucuronidases in plants to modify the structures of glucuronoxylans has not been yet attempted. Glycoside hydrolase (GH) family 115 α-glucuronidases cleave the internal α-D-(4-*O*-methyl)glucopyranosyluronic acid ((Me)GlcA) from xylans or xylooligosaccharides. In this work, a GH115 α-glucuronidase from *Schizophyllum commune, Sc*AGU115, was expressed in *Arabidopsis thaliana* and targeted to apoplast. The transgene effects on native xylans’ structures, plant development, and lignocellulose saccharification were evaluated and compared to those of knocked out glucuronyltransferases *At*GUX1 and *At*GUX2.

**Results:**

The *Sc*AGU115 extracted from cell walls of Arabidopsis was active on the internally substituted aldopentaouronic acid (XUXX). The transgenic plants did not show any change in growth or in lignocellulose saccharification. The cell wall (Me)GlcA and other non-cellulosic sugars, as well as the lignin content, remained unchanged. In contrast, the *gux1gux2* double mutant showed a 70% decrease in (Me)GlcA to xylose molar ratio, and, interestingly, a 60% increase in the xylose content. Whereas *Sc*AGU115-expressing plants exhibited a decreased signal in native secondary walls from the monoclonal antibody UX1 that recognizes (Me)GlcA on non-acetylated xylan, the signal was not affected after wall deacetylation. In contrast, *gux1gux2* mutant was lacking UX1 signals in both native and deacetylated cell walls. This indicates that acetyl substitution on the xylopyranosyl residue carrying (Me)GlcA or on the neighboring xylopyranosyl residues may restrict post-synthetic modification of xylans by *Sc*AGU115 *in planta*.

**Conclusions:**

Active GH115 α-glucuronidase has been produced for the first time in plants. The cell wall–targeted *Sc*AGU115 was shown to affect those glucuronate substitutions of xylan, which are accessible to UX1 antibody and constitute a small fraction in Arabidopsis, whereas majority of (Me)GlcA substitutions were resistant, most likely due to the shielding by acetyl groups. Plants expressing *Sc*AGU115 did not show any defects under laboratory conditions indicating that the UX1 epitope of xylan is not essential under these conditions. Moreover the removal of the UX1 xylan epitope does not affect lignocellulose saccharification.

**Electronic supplementary material:**

The online version of this article (doi:10.1186/s12896-015-0154-8) contains supplementary material, which is available to authorized users.

## Background

The foreseeable shortage of fossil fuels has driven the search for alternative supplies for energy and plastics. Plant biomass, as a renewable natural resource, has the potential to become a feedstock for conversion into fuels, chemicals, and materials. However, harnessing the benefits from the structurally complex plant cell walls remains a major challenge [1,2]. Secondary cell walls, the major component of plant biomass, are primarily composed of cellulose microfibrils embedded in a matrix of hemicelluloses and lignin [3]. The interplay between the biomolecules [4,5] determines the strength of the lignocellulose that inherently contributes to recalcitrance against deliberate extraction or enzymatic hydrolysis [1].

*O*-acetylglucuronoxylans (AcGXs) are the most abundant hemicelluloses present in the secondary wall of dicots [6]. The backbones of AcGXs are formed by (1 → 4)-linked β-D-xylopyranosyl (Xyl) units and are substituted by (1 → 2)-linked α-D-(4-*O*-methyl)glucopyranosyluronic acid ((Me)GlcA) units every 4-16 Xyl residues [7]. The AcGXs are also highly substituted by acetyl residues at the 2-*O* and/or 3-*O* position of the Xyl units [8-13]. More complex substitutions at AcGXs in dicots are uncommon but may exist, such as an α-D-galactopyranosyl (1 → 2)-linked to MeGlcA found in eucalyptus [14]. AcGXs may also associate with lignin through ester, ether, or glycosidic bonds [15,16].

Cell wall modification via *in planta* engineering can be utilized to design cell wall constituents with increased fermentable sugars, polymer extractability, or to tailor other lignocellulose properties [1,2]. This goal is approachable either by manipulating endogenous biosynthetic genes or by expressing microbial polysaccharides-modifying enzymes in plants. Although mutating the endogenous genes related to the xylan backbone [17-19] or the reducing end sequence synthesis [20-22] has impaired plant growth, partially disrupting the side groups, for example, (Me)GlcA [23,24] or *O*-acetylation [25,26], did not seem to affect the fitness of the plant. This tolerance of plants to an altered polymer structure is exemplified by the double knockout of two glucuronosyltrasferases (GlcATs), GUX1 and GUX2, in Arabidopsis, resulting in an almost complete lack of (Me)GlcA substitution in the AcGXs, without affecting the growth of the mutants. Xylan extraction, however, was enhanced in the *gux1gux2* mutants [23].

*In planta* expression of a microbial enzyme offers two advantages. The exogenous enzymes can be chosen to target specific linkages in wall polysaccharides, thus affecting polymer properties in a controlled manner. It can also offer a cost-saving strategy for producing and storing lignocellulolytic enzymes in plants [27-32]. Endo-1,4-β-xylanases (EC 3.2.1.8) are the dominant enzymes that cleave the backbone of AcGXs, while α-glucuronidases (EC 3.2.1.139) and acetyl xylan esterases (EC 3.1.1.72) are the accessory enzymes that remove the (Me)GlcA and acetyl residues, respectively. Several cases have reported the expression of endoxylanases in plants, either as cell wall targeted [28-30] or intracellular enzymes [27,29,31]. Xylans were solubilized better in endoxylanase-expressing plants indicating a potential route for the enhanced extractability of xylans and improved saccharification [28].

Expression of side group–modifying enzymes such as the *Aspergillus nidulans* acetyl xylan esterase (*An*AXE), feruloyl esterase (*An*FAE), α-arabinofuranosidase/β-xylosidase (*An*XA), and the *Xanthomonas oryzae* α-arabinofuranosidase (*Xo*AF) in Arabidopsis [33,34] has been successful without harming plant growth. Whereas, the expression of *Phanerochaete carnosa* glucuronyl esterase (*Pc*GCE) in Arabidopsis [35] and aspen [36] induced morphological changes. Cell wall digestibility was mildly improved in *An*FAE-, *An*XA-, *Xo*AF and *Pc*GCE-expressing plants, but not in *An*AXE-expressing plants although the acetyl content was moderately decreased. As acetylation affects association between xylan chains as well as adsorption to cellulose fibrils, deacetylation may result in tighter interactions of the xylan chains and cellulose in the cell wall, and thus limit the digestibility.

**Figure 1:**
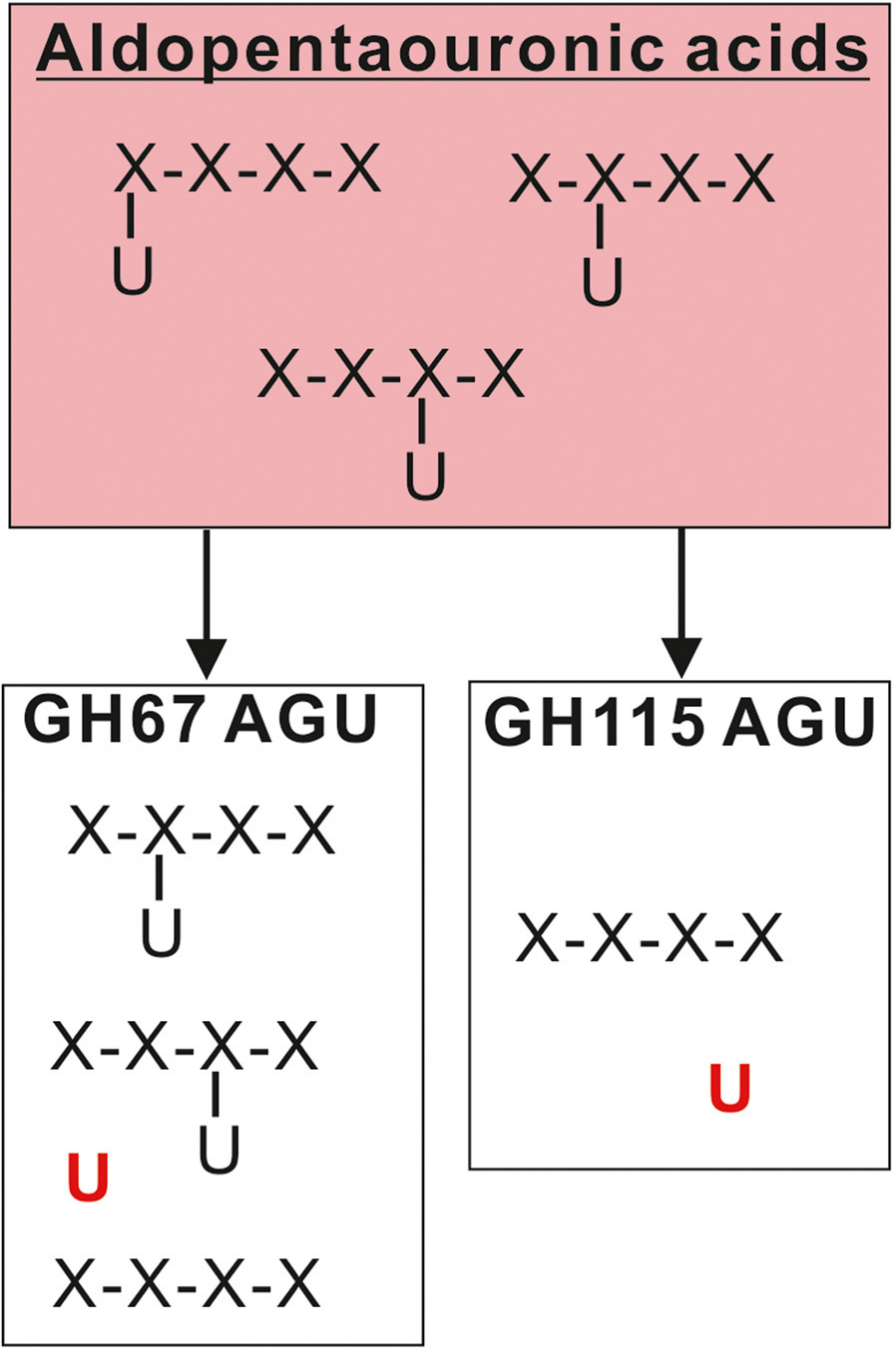
An illustration showing enzymatic actions of two α-glucuronidase families, glycoside hydrolase (GH)67 and GH115, acting on aldopentaouronic acid isomers. GH67 α-glucuronidase cleaves only the (Me)GlcA residue substituted on the nonreducing end of xylotetraose, while GH115 α-glucuronidase acts on terminally and internally substituted (Me)GlcA residues. AGU, α-glucuronidase; X, Xyl; U, (Me)GlcA.

*In planta* expression of the α-glucuronidase targeting the α(1 → 2)-linkage between (Me)GlcA residue and the AcGX backbone has not been attempted. Two glycoside hydrolase families, that is, GH67 and GH115, harbor α-glucuronidases that act either uniquely on the terminal (nonreducing end) or on the internal and terminal (Me)GlcA, respectively (Figure 1) [37-40]. GH115 α-glucuronidase from the white rot fungus *Schizophyllum commune* is known to be active on xylan polymers [40], and its gene sequence has been recently unveiled [41]. In this report, we show that the *S. commune* GH115 α-glucuronidase, codon optimized for expression in plants, can be produced in Arabidopsis as an active enzyme. Biochemical analyses of the overexpressors’ cell walls showed that the *in muro* AcGXs, however, were largely immune to the expressed enzyme.

## Results

### Generation of transgenic arabidopsis lines expressing the *Sc*AGU115 α-glucuronidase in cell walls

The nucleotide sequence of *Sc*AGU115 α-glucuronidase was optimized for plant codon usage, because rare codons could reduce the efficiency of translation or even disengage in the translational machinery [42]. Consequently, the percentage of low frequency (<30%) codons was decreased from 3% in the original sequence to zero in the synthetic gene. The resulting cDNA was cloned under the cauliflower mosaic virus 35S promoter, and the native signal peptide was replaced by the signal peptide from the aspen cellulase *Ptt*CEL9B3 to drive the protein to the apoplast [43,44]. The nucleotide sequence used in the overexpression vector is given in Additional file 1: Figure S1.

**Figure 2:**
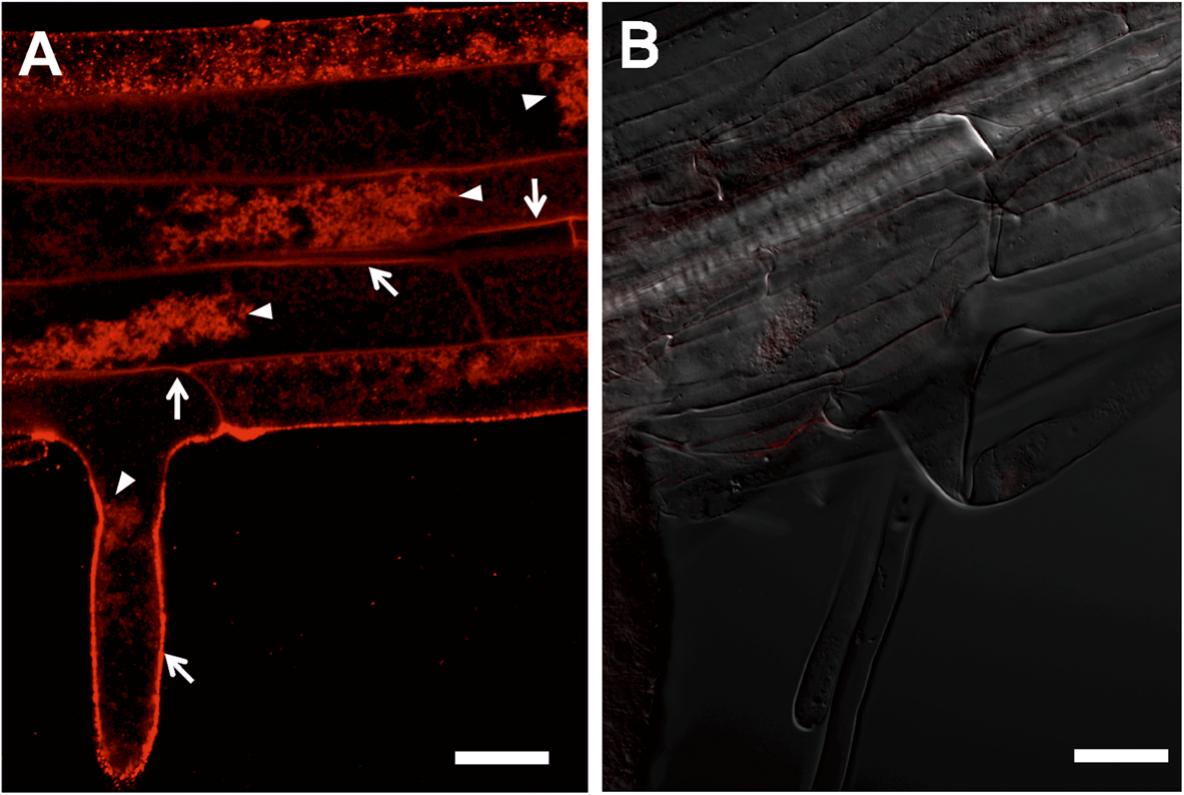
Immunolabeling of the *Sc*AGU115:eGFP fusion protein in the plasmolyzed root cells of the Arabidopsis seedling. **(A)** GFP localization. The shrunk protoplast is shown with an arrowhead. The arrow indicates the signal visible in the cell wall. **(B)** Negative control without primary antibody. Bar = 20 μm.

To verify if the aspen cellulase signal peptide effectively targeted the fusion protein to the apoplast in Arabidopsis, a 35S::*Sc*AGU115:green fluorescent protein (GFP) construct was cloned and expressed in Arabidopsis for protein localization analysis. Since the signal from the GFP was weak and labile in the cell walls due to the low pH [45,46], the fusion protein detected instead using the anti-GFP antibody. This produced a stable signal that was visible mostly in cell walls, even after the protoplasts were plasmolyzed (Figure 2). The signal was also detected in protoplasts, likely corresponding to the protein in transit to cell walls. The aspen signal peptide was functional in Arabidopsis, suggesting that the *Sc*AGU115 was also secreted to the cell wall and potentially deployable for post-synthetic modification of the cell wall.

Transformation of Arabidopsis with 35S::CEL9B3SP:*Sc*AGU115 resulted in 15 independent homozygotic lines that contained a single insert as indicated by the segregation analysis in T2, and 14 of those lines expressed the transgene, as found with RT-PCR (Additional file 2: Figure S2A).

### Extracts of transgenic lines expressin*g Sc*AGU115 exhibit enzymatic activity hydrolyzing internal (Me)GlcA side groups of xylan

The protein extracts from the inflorescence stems of the transgenic plants were assayed for α-glucuronidase activity using a commercial test kit. The substrate used in the kit is a mixture of aldotriouronic, aldotetraouronic, and aldopentaouronic acids (2:2:1) obtained by acid hydrolysis of glucuronoxylans, resulting in either internal or terminal MeGlcA substitution. Thus, this assay measures GH67 and GH115 types of activity. Background α-glucuronidase activity was observed in the wild type (WT) and was most likely due to endogenous Arabidopsis NADH that was co-extracted and interfered with the detection (Additional file 2: Figure S2B). Only 20% of the total number of transgenic lines that express the construct showed α-glucuronidase activity above the WT level (Additional file 2: Figure S2B). The low number of transformants showing α-glucuronidase activity was likely caused by the constitutive promoter, which may have triggered counteractive responses in plants, as has been seen in tall fescue expressing *Trichoderma reesei* endoxylanase [29].

**Figure 3:**
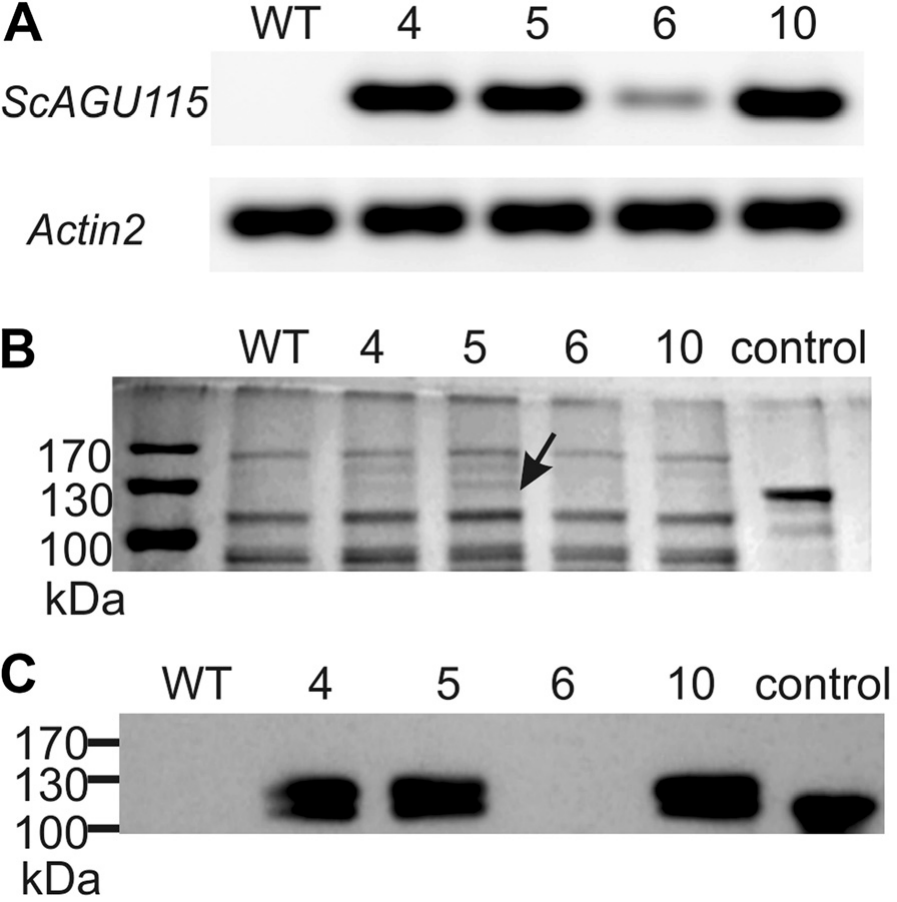
*Sc*AGU115 gene expression and protein analysis in Arabidopsis transgenic and WT plants. **(A)** RT-PCR analysis of *ScAGU115* expression. Fragments of *ScAGU115* (315 bp) and *Actin2* (201 bp) were amplified from total RNA isolated from the stem tissues. **(B)** The soluble protein was separated on SDS-PAGE and visualized with Coomassie staining. The arrow indicates the novel protein bands that are visible on lines 4, 5, and 10. **(C)** The soluble proteins were immunoblotted with anti-*Sc*AGU115 primary antibody. Control, *Sc*AGU115 native enzyme [40].

The three lines that showed α-glucuronidase activity (lines 4, 5, and 10), and one line (6) that was transgenic but did not show a detectable level of α-glucuronidase activity were selected for further analysis and re-grown. RT-PCR analysis, performed on the re-grown lines, showed that the levels of the *Sc*AGU115 transcripts were higher in lines 4, 5, and 10 and lower in line 6 (Figure 3A), which was consistent with the earlier activity assays. The sodium dodecyl sulfate-polyacrylamide gel electrophoresis (SDS-PAGE) analysis of soluble proteins extracted from the stems of the transgenic and WT plants revealed the presence of two novel protein bands in lines 4, 5, and 10, which were not detected in the WT and in line 6 (Figure 3B). Western blot analysis using the anti-*Sc*AGU115 antibody confirmed that the novel bands contained *Sc*AGU115, since signals were detected in lines 4, 5, and 10 (Figure 3C), and after longer exposure in line 6 (data not shown). The lower molecular weight protein band in the transgenic lines migrated close to the native *Sc*AGU115 protein (control) at 125 kDa [40], indicating the glycosylation level in Arabidopsis closely resembled that of the native fungus. The higher molecular weight protein band may represent another form of processing in Arabidopsis.

**Figure 4:**
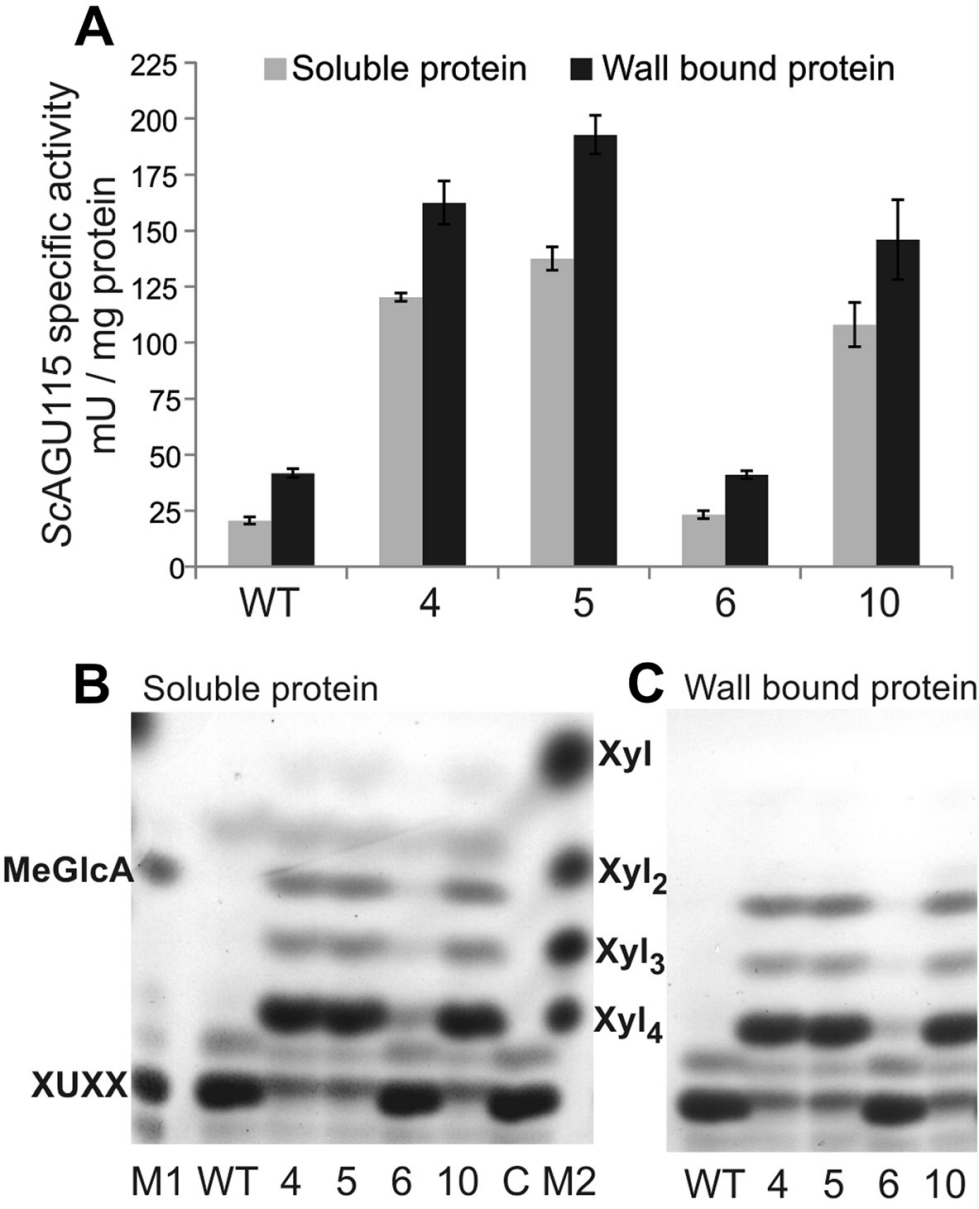
Analysis of *Sc*AGU115 enzyme activity in Arabidopsis transgenic and WT plants. **(A)** The soluble and wall-bound proteins were analyzed for α-glucuronidase activity using a commercial assay. The error bar represents the standard deviation of three biological replicates. **(B)** and **(C)** TLC analysis of XUXX incubated with soluble **(B)** or wall-bound **(C)** protein. XUXX, a xylotetraose carrying MeGlcA at penultimate xylose from the nonreducing end; C, water blank; M1, marker for XUXX and MeGlcA; M2, marker for xylose (Xyl), xylobiose (Xyl_2_), xylotriose (Xyl_3_), and xylotetraose (Xyl_4_).

To test if the expressed protein was active, α-glucuronidase activity was determined from the soluble proteins extracted from the inflorescence stems. The specific α-glucuronidase activity in the soluble proteins extracted from lines 4, 5, and 10 was about five to six fold higher than the background WT level, whereas no activity above the WT level was detected in line 6 (Figure 4A). Activity was also detected in wall-bound proteins, at an even higher level than in the soluble fractions (Figure 4A), confirming that there were significant amounts of wall-bound *Sc*AGU115 enzyme in the transgenic lines.

To test more specifically for the AGU115 type of activity, the soluble extracts from Arabidopsis were further analyzed for their activity toward the internally substituted aldopentaouronic acid XUXX. Thin layer chromagraphy (TLC) analysis clearly showed that the XUXX substrate was degraded to xylotetraose and MeGlcA by lines 4, 5, and 10 (Figure 4B). Only a faint band corresponding to xylotetraose was produced by line 6. No apparent degradation of XUXX was observed in the WT extract or water blank (Figure 4B) or by a commercial GH67 enzyme tested as a comparison (data not shown). Xylotriose was also detected in the hydrolyzates of lines 4, 5, and 10, suggesting that the formed xylotetraose is further degraded to shorter oligosaccharides presumably by an endogenous β-xylosidase or endoxylanase likely to be present in crude protein samples [47]. The enzymatic activity of the wall-bound proteins was also tested against the XUXX substrate, and similar degradation profiles were observed (Figure 4C). In summary, lines 4, 5, and 10 expressed *Sc*AGU115 in an active form, in the apoplast, and in relatively high amounts.

### Plant growth and development are not compromised by expression of active *Sc*AGU115

**Figure 5:**
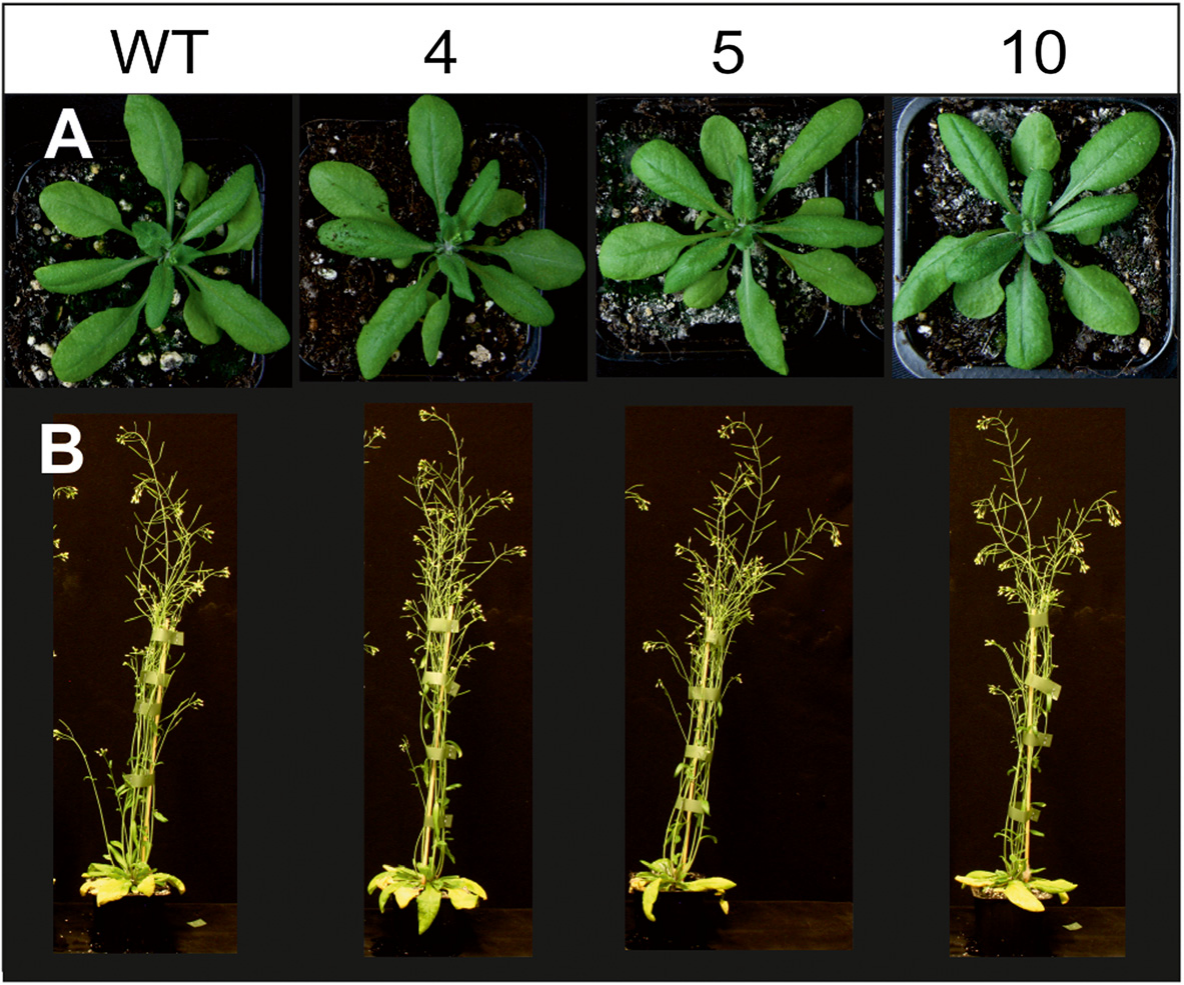
Morphology of transgenic and WT plants grown under long-day conditions for 7 weeks. The **(A)** rosette size and **(B)** plant height were not affected by the expression of active *Sc*AGU115.

Morphology of the transgenic plants (lines 4, 5, and 10) grown under long-day conditions was examined to test if the expression of *Sc*AGU115 affected plant growth and development (Figure 5). There was a possibility that the AGU activity could induce changes in cell walls leading to morphological changes or that certain motifs in the foreign protein could be perceived as pathogen-related molecular patterns (PAMPs) triggering defense responses that slow the growth [48,49]. No differences in plant height or rosette diameter were observed (Figure 5). The morphology of the plants was similar to those of the WT, and no obvious stress responses, such as poor growth, or leaf necrosis, or senescence, were observed.

### Cell wall chemistry and integrity are not affected in the Arabidopsis transgenic lines that exp*ress Sc*AGU115

**Figure 6:**
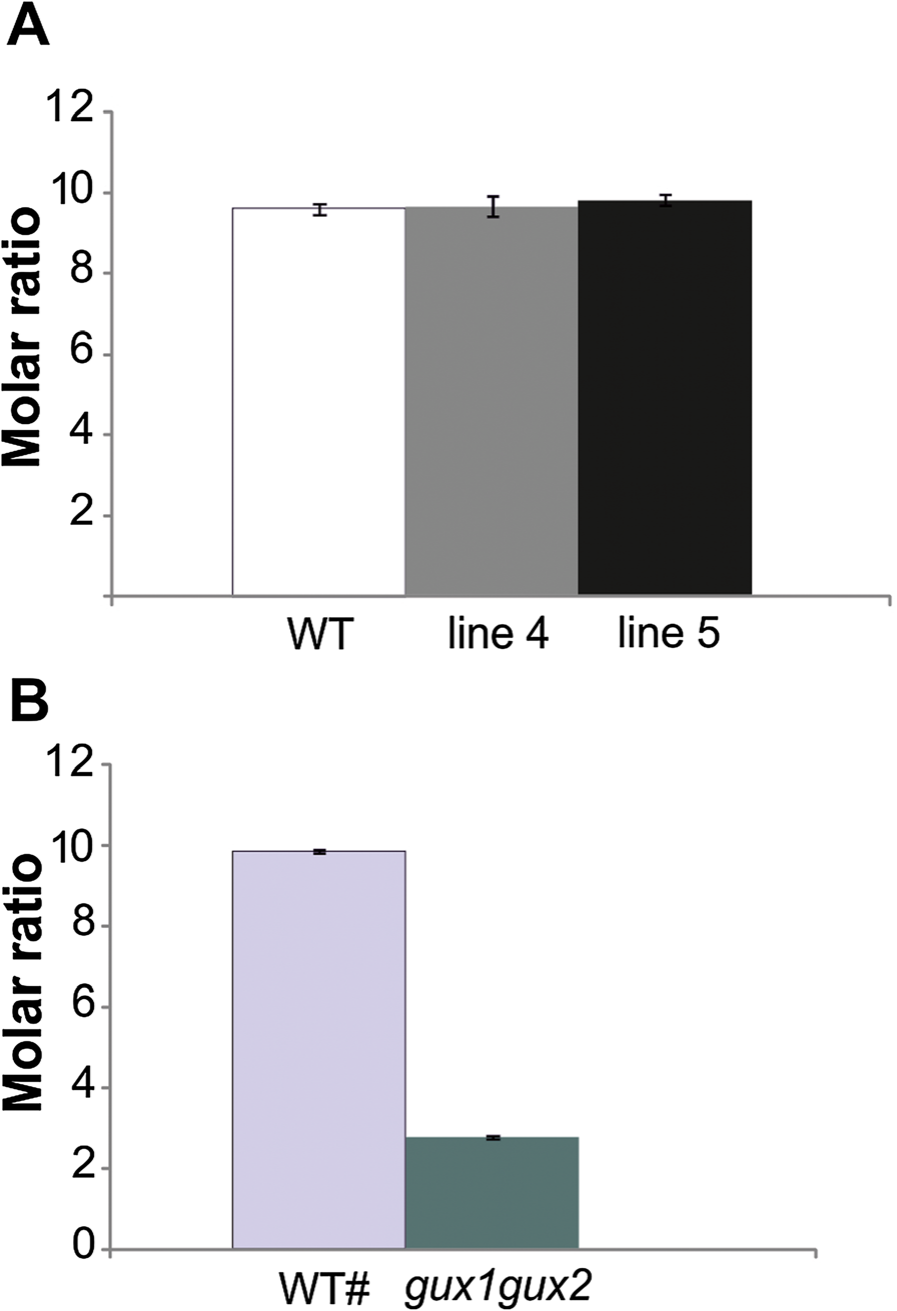
Molar ratio of MeGlcA to Xyl (per 100 xylose residues) in the stem tissues of Arabidopsis. Xyl and (Me)GlcA were liberated from the AIRs of the stem tissues with acid methanolysis and analyzed with gas chromatography. **(A)** Comparison of the *Sc*AGU115 transgenic and WT plants. The error bar represents the standard deviation of five biological replicates. **(B)** Comparison of the *gux1gux2* and WT# plants. The error bar represents the standard deviation of three technical replicates from a pool of 30 plants.

Since the recombinant *Sc*AGU115 possesses activity toward the internal MeGlcA, we were interested in how the enzyme acts on the native AcGX polymers within the cell wall. The alcohol insoluble residues (AIRs) of the inflorescence stem tissues were examined for chemotypic changes caused by the expression of *Sc*AGU115 in the apoplast. The analyses were performed only on lines 4 and 5 that produced the highest levels of the recombinant enzyme. The analysis of non-cellulosic sugars showed that there were no changes in the (Me)GlcA or other sugar components in lines 4 and 5 compared to the WT (Table 1). This resulted in similar molar ratios of (Me)GlcA to Xyl in the transgenic and WT plants (Figure 6A). As a comparison, we studied the Arabidopsis *gux1gux2* double mutant in which the two endogenous GlcATs, GUX1 and GUX2, were disrupted [23]. A significant decrease in total (Me)GlcA content was detected in *gux1gux2* (Table 1), and as a result, the molar ratio of (Me)GlcA to Xyl was decreased by 70% compared to the WT (Figure 6B). Notably, the amount of Xyl and Man was increased by 59% and by 30%, respectively, in *gux1gux2* compared to the WT, whereas no changes in Xyl or Man levels were detected in the *Sc*AGU115 transgenic lines (Table 1).

**Table 1:** **Non-cellulosic sugar contents (% of de-starched AIRs) in the inflorescence stem tissues of Arabidopsis transgenic (lines 4, 5) and WT plants**

**Genotype**	**Ara**	**Rha**	**Xyl**	**Man**	**Gal**	**Glc**	**GalA**	**MeGlcA**	**GlcA**
WT	1.3 ± ^a^0.0	1.7 ± 0.1	14.1 ± 0.9	1.3 ± 0.0	1.6 ± 0.0	2.6 ± 0.1	6.6 ± 0.1	1.2 ± 0.1	0.7 ± 0.1
line 4	1.4 ± 0.1	1.7 ± 0.1	13.2 ± 0.6	1.2 ± 0.1	1.6 ± 0.1	2.5 ± 0.1	6.6 ± 0.3	1.1 ± 0.1	0.7 ± 0.1
line 5	1.2 ± 0.2	1.6 ± 0.1	13.1 ± 0.8	1.2 ± 0.1	1.5 ± 0.1	2.5 ± 0.2	6.5 ± 0.4	1.1 ± 0.0	0.7 ± 0.1
WT#	1.2 ± ^b^0.1	1.2 ± 0.1	14.8 ± 1.1	1.3 ± 0.1	1.6 ± 0.1	2.5 ± 0.3	4.3 ± 0.3	1.1 ± 0.1	1.0 ± 0.1
*gux1gux2*	1.3 ± 0.4	1.4 ± 0.3	23.5 ± 0.9*	1.7 ± 0.1*	1.8 ± 0.4	2.4 ± 0.4	4.7 ± 0.9	0.6 ± 0.0*	0.4 ± 0.0*

The (Me)GlcA-deficient mutant, *gux1gux2* [23], was also analyzed with the corresponding wild-type (WT#) plants.

a, the ± represents the standard deviation of five biological replicates; b, the ± represents the standard deviation of three technical replicates from a pool of 30 plants; *indicates significance different from WT# (*t* test, p value < 0.05); Ara, arabinose; Rha, rhamnose; Xyl, xylose; Man, mannose; Gal, galactose; Glc, glucose; GalA, galacturonic acid; MeGlcA, 4-*O*-methyl-glucuronic acid; GlcA, glucuronic acid.

To further examine the xylan structure in the transgenic plants in detail, AcGX was extracted with DMSO after mild delignification, and the structure was analyzed with heteronuclear single quantum coherence (HSQC) nuclear magnetic resonance (NMR) spectroscopy [9]. With this method, it is possible to distinguish nonacetylated and acetylated Xyl residues, including the ones carrying the (Me)GlcA substituent, and determine their relative content. No differences in the content of any of the xylan structural elements, including acetylated (X3G2) and nonacetylated (XG2) MeGlcA-Xyl, were detected in the AcGX samples isolated from lines 4 and 5 compared to the WT (Additional file 3: Figure S3). In contrast, a drastic decrease in the (Me)GlcA substituted Xyl was clearly detected in *gux1gux2* [9].

The guaiacyl (G), syringyl (S), and *p*-hydroyphenyl (H) lignins and the carbohydrate fraction in the transgenic and WT plants were examined via pyrolysis-gas chromatography/ mass spectrometry (Py-GC/MS) (Additional file 4: Table S1). The S and G lignins remained unchanged, while the H lignin was slightly increased by 4% in line 4 (*t* test, p < 0.05), possibly as a stress reaction in this particular line [50]. The carbohydrate fraction was unchanged between the samples, indicating the main constituents of the cell wall were not affected by *Sc*AGU115 expression. This is consistent with the comparable non-cellulosic sugar content obtained in the sugar analysis.

**Figure 7:**
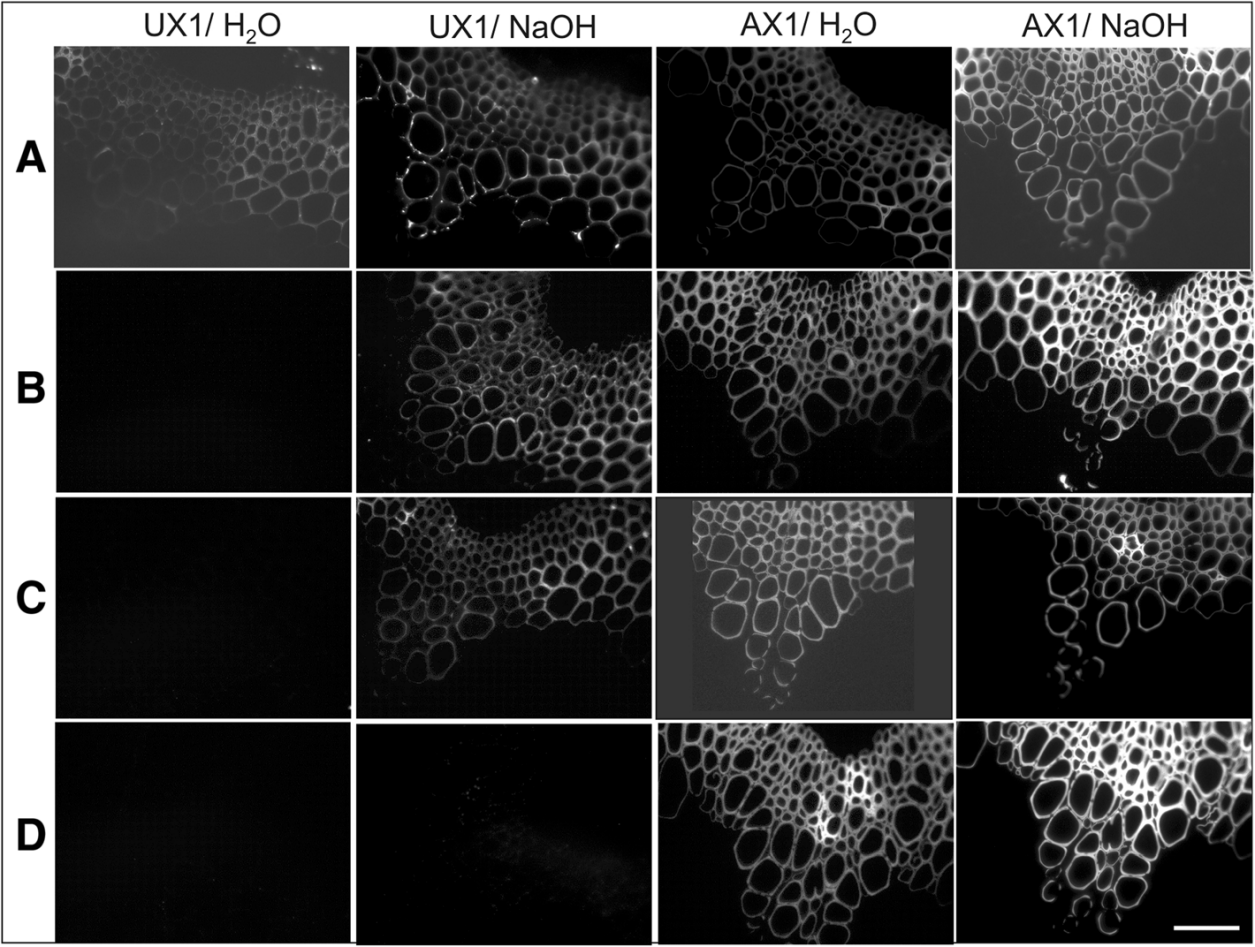
Immunolabeling of the basal stem sections with UX1 and AX1 antibodies. The 0.5 μm sections of WT **(A)**, transgenic plants line 4 **(B)** and line 5 **(C)**, and the (Me)GlcA-deficient mutant, *gux1gux2* **(D)**. The sections were pretreated either with 0.05 M NaOH overnight before the labeling in order to reveal antibody binding to de-acetylated samples or with water for the same duration to analyze the antibody binding in the acetylated samples. Note that UX1-reactive sites in native samples were absent in *Sc*AGU115 expressing lines. Bar = 50 μm.

To visualize the distribution of the (Me)GlcA residues in the stem sections of the transgenic and WT plants, we labeled the basal stem sections using the (Me)GlcA specific antibody UX1 [51]. A weak signal was evident in the water-treated WT (Figure 7A), especially in the interfascicular fibers, but it was missing in the water-treated samples of line 4 (Figure 7B) and line 5 (Figure 7C). In the alkaline-treated stem sections, where esterified acetyl residues were removed, the signal was strongly visible in all samples, and the signal strength was the same for the transgenic and WT plants. Acetylation is known to restrict the binding of UX1 to (Me)GlcA residues [51]. No signal was detected in either the water- or alkaline-treated *gux1gux2* sections (Figure 7D). The water- and alkaline-treated sections were labeled with the xylan-specific AX1 antibody [52], indicating that xylan was present and accessible to the antibodies in all samples. The disappearance of UX1 labeling in the water-treated *Sc*AGU115 shows that all UX1-accessible (Me)GlcA residues, which are present in small amounts in the WT and most likely are situated in the regions of xylan that are not acetylated, were cleaved in the transgenic lines.

Finally, to test if wall digestibility was affected by the removal of *Sc*AGU115 accessible (Me)GlcA moieties, the main inflorescence stems of the transgenic (lines 4 and 5) and WT plants were subjected to hot water or 0.5 M NaOH pretreatments, after which the stems were hydrolyzed with a mixture of commercial lignocellulose-degrading enzymes. There was no difference in the reducing sugars released from the stem tissues by the enzymes after any of these pretreatments (Additional file 5: Figure S4), which shows that cell wall digestibility in the *Sc*AGU115 transgenic plants did not change.

## Discussion

Codon-optimized *Sc*AGU115 α-glucuronidase was produced in an active form in Arabidopsis without altering plant development. The recombinant enzyme showed activity against internal (Me)GlcA substitutions of non-acetylated aldopentaouronic acid. However, no decrease in the (Me)GlcA content or improved digestibility of the stem tissues was observed despite the high reactivity of *Sc*AGU115 towards the non-acetylated hardwood xylan polymer *in vitro* has been detected [40]. The lack of quantitatively detectable change in the (Me)GlcA content (Figure 6) or (Me)GlcA substituted Xyl*p* units (Additional file 3: Figure S3) in the xylans of *Sc*AGU115 expressing lines compared to the wild type indicates that the action of this enzyme is limited *in planta*. Therefore, it appears that reducing the (Me)GlcA content by mutating the endogenous GlcATs, GUX1 and GUX2, is more efficient than post-synthetic modification with an exogenous GH115 α-glucuronidase. Interestingly, *gux1gux2* showed, in addition to a significantly reduced (Me)GlcA content, an elevated amount of xylan and mannan as indicated by the increased Xyl and Man content (Table 1). The increase in Xyl levels in *gux1gux2* was similarly observed by [24]. This may suggest a competing utilization of UDP-GlcA in the double mutant as the substrate for UDP-GlcA decarboxylase to produce UDP-Xyl [53].

The unchanged levels of (Me)GlcA, in spite of active AGU115 being expressed in the apoplast, are most likely caused by the acetylation in native GX. Arabidopsis GX is known to be highly acetylated. As many as 50% of the Xyl residues are either mono- or di-acetylated [9,13]. Furthermore, of the (Me)GlcA-substituted Xyl residues, only 23% are non-acetylated whereas 77% carry acetylation at the 3-*O* position [9]. Moreover, the pattern of acetylation is rather uniform along the GX chain. Most acetyl groups are located in every other Xyl residue [9,54], and occasionally, on two adjacent Xyl residues [9]. Therefore the sites where the acetyl groups are absent in several adjacent residues are probably very scarce. Since the presence of acetylation changes the hydrophobicity of xylans by replacing the hydroxyl group with the more hydrophobic acetyl, dense acetylation would expectedly affect the enzyme-substrate binding. In GH115 active sites, in addition to the important +1 subsite, the neighboring Xyl residues at the reducing end side (+R2, +R3) (nomenclature according to [55]) and the nonreducing end side (+NR2) of the xylan chain may be crucial for productive enzyme-substrate binding, especially since the enzyme is active on the internal MeGlcA substitutions [56,57]. Thus, despite their small size, the acetyl residues linked at or close to the (Me)GlcA substituted Xyl may interfere with productive substrate binding. The hindering effect of acetyl substitution on *Sc*AGU115 action has been shown *in vitro* since only 10% of the theoretical MeGlcA was released from birch AcGX compared to the 49% yield from deacetylated GX [40]. Thus, xylan acetylation is expectedly the key parameter restricting the action of GH115 enzymes in native cell walls. Further work is needed to test the activity of this family of α-glucuronidases using model substrates with well-defined acetylation patterns.

The proposed shielding of (Me)GlcA substituents by acetylation is consistent with the immunolocalization results for the UX1 antibody (Figure 7). A small but clear difference between the transgenic and WT plants in UX1 antibody binding to native cell walls, and no difference in UX1 binding after cell wall deacetylation was observed. Thus, the UX1 antibody marks the *Sc*AGU115 accessible epitodes on native AcGX, which is known to be very low abundant in Arabidopsis [9]. Although we did not detect a change in the content of the non-acetylated (Me)GlcA substituted Xyl in the DMSO-solubilized AcGX of transgenic plants with HSQC NMR, the signal from these residues in the WT was very low (Additional file 3: Figure S3). Moreover, the method extracts only ca. 20% xylan [9]. Therefore, we propose that the UX1 labeling provided a more sensitive detection of the rare *Sc*AGU115 accessible sites, revealing the action of this enzyme *in planta* (Figure 7).

The *in planta* modification of (Me)GlcA is, nonetheless, an attractive approach for better extractability of xylans as has been demonstrated with *gux1gux2* [23]. However, the structural heterogeneity of AcGX may not permit a single hydrolytic enzyme to work effectively. If the *in planta* modification of (Me)GlcA content by GH115 α-glucuronidase is hindered by xylan acetylation, a viable approach could be co-expression with an acetyl xylan esterase (AXE) to obtain synergism between these side-group removing enzymes. Production of the carbohydrate esterase (CE) family 1 AXE in plants has been attempted, and the transgenic plants showed, similarly as with *Sc*AGU115, no visible morphological changes and no effect on saccharification [33]. No improvement in saccharification was observed in *gux1gux2* hydrolyzed without pretreatment [24], even though the extractability of xylan was improved [23], which shows that a strong decrease in (Me)GlcA substitution by mutating endogenous GlcATs might not be optimal for biomass improvement for saccharification. Therefore, the co-expression of AGU115 and AXE in plants could be a strategic approach for designing a wall amenable to better xylan extractability and saccharification.

Nonetheless, the resistance of AcGX to *Sc*AGU115 *in planta*, combined with the lack of adverse effects of the transgene on plants, opens up the possibility of using such transgenic plants to produce the active enzyme for biotechnological applications. For example, a thermostable variant expressed in biofuel feedstocks could be envisaged for removing hemicellulose more efficiently during pretreatment and for reducing enzyme loads during enzymatic hydrolysis. Thermostable xylanases have been engineered and expressed in plants, but these enzymes were also active in ambient conditions *in planta* [28]. To our knowledge, the *Sc*AGU115 is the first enzyme that does not appreciably change the cell wall *in planta* while exhibiting normal activity when extracted.

## Conclusion

In this work, active GH115 α-glucuronidase from Schizophyllum commune was produced for the first time in plants. The ScAGU115 expressing Arabidopsis thaliana were viable and no morphological changes were observed. However, only low amount of cell wall (Me)GlcA substituents, which were recognized by anti-UX labeling of native xylans, was removed by the active enzyme. Moreover, the ScAGU115 expression did not improve the saccharification efficiency of the transgenic plants. The limited action of ScAGU115 in planta is envisaged to be due to shielding effects of acetyl substituents on xylans. Therefore, co-expressing the GH115 α-glucuronidase with an acetyl xylan esterase can be a strategic approach to modify the side groups of cell wall xylans.

## Methods

### Optimizing plant codon usage and cloning of the *Sc*AGU115 *α-glucuronidase cDNA* sequence in Arabidopsis

The full-length cDNA encoding *Sc*AGU115 α-glucuronidase (GenBank accession ADV52250.1) [41] was codon-optimized for expression in Populus, and the native signal peptide sequence was replaced with hybrid aspen cellulase *Ptt*CEL9B3 (GenBank accession AY660968.1; [43]) and flanked by attB1 and attB2 recombinant sites. The synthetic gene (GenScript (http://www.genscript.com) was recombined into the pDONR207 vector as an entry clone using the Gateway System (Invitrogen, Life Technologies), sequenced, and cloned into the destination vector, which was pK2WG7.0 for overexpression in Arabidopsis with a stop codon or pK7FWG2.0 for overexpression as the GFP-fusion protein for intracellular protein localization [58]. The *Arabidopsis thaliana* (Columbia ecotype; Col-0) transformation was performed via *Agrobacterium tumefaciens* (GV3101) infiltration using the floral dip method [59].

### Gene expression screening of the homozygotic lines

Fifteen homozygotic lines were obtained, and 10 plants for each line and the WT were grown in a 16 h photoperiod with 120 μmol m^−2^ s^−1^ light intensity at 23°C for 7 weeks. Basal 10 cm of the main stem and 5 cm of side shoots were dissected and divided into three biological replicates. The collected samples were frozen in liquid nitrogen before storage at –80°C. The plant tissues were ground in a liquid nitrogen bath using a mortar and pestle. The ground tissue was used for RNA isolation and protein extraction. Total RNA was isolated using TRIzol Reagent (Life Technologies) and treated further with DNase (DNA-free Kit, Ambion) to remove any potential genomic DNA contamination. A 1 μg RNA sample was used to synthesize cDNA (iScript cDNA Synthesis Kit, Bio-Rad), and 2 μl of the cDNA diluted 1:4 was used for PCR amplification. The gene-specific primers designed for amplification of *ScAGU115* were SP5(F), 5’-tggaatgcacagggtatga-3’ and R1(R): 5’-caagcaccaacgtgcacggactcaggtgg-3’; and the expected size of the transcript product was 315 bp. Amplification of *Actin2* (At3g18780) was used as the internal control, using the primers ACT1(F), 5’-ggtaacattgtgctcagtggtgg-3’and ACT3(R): 5’-ctcggccttggagatccacatc-3’; and the expected size for the transcript product was 201 bp. The forward primer designed for amplification of the *Actin2* was intron spanning; thus, genomic DNA contamination in the sample was detectable by the appearance of a DNA band at 291 bp.

### Protein extractions

An aliquot of 300 μl buffer A (0.2 M sodium succinate, 10 mM CaCl_2_ and 1% (*w/v*) PVPP, pH 5.5) [60] was added into 100 mg ground stems, shaken at 4°C for 2 h and spun at 14,000 rpm for 15 min. The supernatants were collected as soluble fraction proteins. The extraction was continued by adding 200 μl of buffer B (0.2 M sodium succinate, 10 mM CaCl_2_, and 1 M NaCl, pH 5.5) into the residue to liberate the wall-bound proteins. The mixtures were shaken at 4°C for 30 min, followed by centrifugation as above, and yielded wall-bound protein fractions.

### *Sc*AGU115 α-glucuronidase activity assay

Protein concentrations were determined according to the Bradford method [61] and adjusted to 0.6 and 0.2 mg/ml for the soluble and wall-bound fractions, respectively. α-Glucuronidase activity assay was performed with 16 μg of protein using the Megazyme kit (Megazyme, Ireland; K-AGLUA).

### Western analysis

Aliquots of soluble protein samples equivalent to 12 μg proteins were separated on SDS-PAGE alongside 60 ng of the native *Sc*AGU115 α-glucuronidase [40]. Western analysis was performed as described by [61]. The proteins were blotted on the PVDF membrane and labeled with primary antibody (1:2000) raised in rabbit against the native *Sc*AGU115 α-glucuronidase [41]. The secondary antibody (1:10,000) was horseradish peroxidase conjugated anti-rabbit IgG (GE Healthcare, NJ, US), and the detection was done with Amersham ECL luminol reagents (GE Healthcare).

### TLC analysis of *Sc*AGU115 α-glucuronidase hydrolyzates

The α-glucuronidase activity in the protein samples was analyzed against the aldopentaouronic acid substrate, XUXX [51]. XUXX (30 μg) was treated with 12 μg soluble proteins in extraction buffer A at 37°C overnight. The hydrolyzates were examined with TLC following the procedures described in [60]. Briefly, 2 to 5 μl of the standards or samples were spotted on Silica gel 60 TLC plates (Merck) and developed in a butanol/acetic acid/water (2:1:1) solvent/aqueous mixture. For staining, the plates were dipped in a solution of 0.5% (*v/v*) thymol and 5% (*v/v*) H_2_SO_4_ in ethanol, dried for 5 min, and then heated at 100°C for 10 min. The plates were scanned within 15 min after staining. Before incubation with the XUXX substrate, the wall-bound proteins were desalted with Microcon centrifugal filters (30,000 MWCO; Millipore) according to the manufacturer recommended procedures, in order to ease the mobility of the hydrolyzates when they were separated on the silica plate. The XUXX was also incubated in buffer A (without PVPP) with an excessive amount of the *Geobacillus stearothermophilus* AGU67 α-glucuronidase (30 U/g XUXX; Megazyme).

### Preparation of alcohol insoluble residues

Thirty plants for each of the transgenic lines that overexpress the 35S:: *Sc*AGU115 construct (lines 4, 5, 6, and 10) and 30 WT plants were grown during a 16 h photoperiod with 120 μmol m^−2^ s^−1^ light intensity at 23°C for 7 weeks. Plant morphology was photographed once a week during weeks 2 to 5. Basal 10 cm of the main stem and 5 cm of side shoots were dissected and divided into five biological replicates. The collected samples were frozen in liquid nitrogen before storage at –80°C. The stems and shoots were freeze-dried and ground using a bead mill (30 Hz, 90 sec), and AIRs were prepared from the ground biomass as described in [62].

### Analysis of non-cellulosic sugars in the stem tissues with acid methanolysis and gas chromatography (GC) analysis

The acid methanolysis procedures were modified from [63] for a small-scale analysis. The starch was removed by *Bacillus licheniformis* α-amylase (Megazyme, 5 U/mg AIR) [62], and 2 mg of the de-starched AIRs was placed in a KIMAX tube. The monosaccharide standards were premixed and dissolved in dry methanol, after which they were aliquoted into three parallel standard series. The standard series for arabinose and rhamnose were 2.5-10-25-40 μg; for galactose, mannose, galacturonic acid, glucuronic acid, and glucose, 5-20-50-80 μg; and for xylose, 50-100-400-800 μg. The monosaccharide standards were dried completely under a stream of nitrogen and dried further in a vacuum oven together with the preweighed de-starched AIRs at 40°C for 20 min. After that, 0.5 ml 2 M HCl/methanol was added to the samples and standards; and the mixtures were incubated at 90°C for 16 h. After the acid methanolysis, the tubes were cooled, and 25 μl of pyridine was added to neutralize the solution. An amount of 100 μg sorbitol was also added as the internal standard. The solution was evaporated completely under a stream of nitrogen; then 300 μl methanol was added, and the solution was evaporated again. The methyl ester methyl glycosides/methyl glycosides were trimethylsilylated and separated by gas chromatography according to the procedures described in [63] and [64], respectively.

### Lignin content analysis by pyrolysis-GC/mass spectrometry

About 50 μg (±10 μg) of the AIRs were added in a pyrolyzer equipped with an autosampler (PY-2020iD and AS-1020E, Frontier Lab, Japan) and connected to a GC/MS (7890A/5975C; Agilent Technologies). After 20 sec of pyrolysis at 450°C, the pyrolyzate was separated on a DB-5 capillary column (30 m long, 0.25 mm i.d., 0.25 μm film thickness; Agilent Technologies) with the following temperature profile: 40°C- 32°C/min- 100°C- 6°C/min- 120°C- 15°C/min- 250°C- 32°C/min- 320°C. The total run time was 19 min, and the mass peaks were scanned in the range of *m/z* 35–250. Peak identification and integration were performed as described by [65] using the manufacturer’s software (Chemstation, Agilent Technologies).

### Saccharification of biomass

Basal 10 cm of the main stem were collected from 10 plants (7 weeks old). Saccharification was performed in a high throughput assay developed by [66]. Briefly, the stems were ground, pretreated either in water or 0.5M NaOH, washed five times in 25 mM NaOAc buffer, pH 4.5, and subjected to 8 h saccharification using 7 FPU/g of a 4:1 mixture of Celluclast and Novozyme 188 (Novozymes, Bagsvaerd, Denmark). After hydrolysis, the reducing sugars were determined using the MBTH colorimetric method as described in [66].

### NMR analysis of AcGX

AcGX was isolated from about 150 mg of de-starched AIRs according to the procedures described in [9]. Before the NMR measurement, the isolated AcGX was exchanged three times in D_2_O. NMR spectra were recorded at 22°C on a 600 MHz Bruker Avance III NMR spectrometer equipped with a Q-CPN cryoprobe. The quantitative HSQC spectra were acquired according to the procedures described in [9].

### Immunolocalization of xylan and (Me)GlcA in basal stem sections

Samples (1 cm segments of basal inflorescence stems) were immersed in a fixative solution (4% paraformaldehyde and 0.05% glutaraldehyde in 25 mM phosphate buffer, pH 7.2). After three washes in 25 mM phosphate buffer, pH 7.2, the segments were dissected into approximately 3-mm-long pieces, dehydrated in a gradient ethanol (30%, 50%, 70%, 80%, 90%, 95%, and 99.5%) series, and embedded in LR White resin according to the manufacturer’s instructions (TAAB, Reading, UK). The incubation at each step was performed in a laboratory rotator. The immunolabeling procedures were performed according to [51] with modifications. Transverse 0.5-μm-thick sections were labeled with UX1 (1:3) and AX1 (1:20) primary antibody diluted in 1% BSA in 0.1 M phosphate buffer, pH 7.2. The secondary antibody was anti-mouse IgG conjugated to Alexa Fluor 647 (Life Technologies) diluted in 1:100 in 1% BSA and 0.05% Tween-20 in phosphate buffer, pH 7.2. The sections were mounted in Milli-Q water, and the fluorescence signal was observed using the excitation filter 646 nm and emission filter 664 nm.

### GFP localization of *Sc*AGU115 protein in Arabidopsis root cell walls

The full-length cDNA fused with GFP was cloned into the binary vector described above. Seven-day-old seedlings of the T2 generation were plasmolyzed in 20% (*v/v*) mannitol and immediately fixed in 2% (*v/v*) paraformaldehyde in MTSB buffer (50 mM PIPES, 5 mM EGTA, 5 mM MgSO_4_, pH 7) and 0.1% (*v/v*) Triton X-100 for 1 h. After washing in water, the seedlings were treated with 100% methanol for 20 min. The methanol concentration was gradually decreased every 2 min until the final concentration reached approximately 20%. Cell walls were digested in 2 mM MES pH5.5 with 0.2% (*v/v*) driselase (Sigma, MO; D9515-1G) and 0.15% (*v/v*) macerozyme R-10 (Duchefa Biochemie, Haarlem, the Netherlands; M8002.0001), and the mixture was incubated at 37°C for 30 min. After washing in water, the membranes were permeabilized with 3% (*v/v*) IGEPAL C630 and 10% (*v/v*) DMSO in MTSB buffer at 37°C for 30 min. The seedlings were washed three times in MTSB buffer and blocked with 3% (*v/v*) BSA in MTSB buffer for 20 min, followed by labeling with the primary rabbit anti-GFP antibody (TorreyPines Biolabs, NJ, US; TP401) diluted in 1:1000 in 1% (*v/v*) BSA in MTSB buffer at 37°C for 2 h. The seedlings were washed two times in MTSB buffer for 5 min followed by incubation with the secondary antibody goat anti-Rabbit IgG H&L conjugated with DyLight 594 (Agrisera, Sweden; AS111815) diluted 1:200 in 1% (*v/v*) BSA in MTSB buffer at 37°C for 1 h. The seedlings were washed two times in MTSB buffer for 5 min and mounted in AF1 anti-fadent medium (Citifluor, London, UK). Fluorescence signals were observed under an Axioplan inverted microscope equipped with a Zeiss LSM780 spectral confocal laser scanning microscope, using a 561 nm laser probe and a 582-670 nm emission filter.

## Additional files

Additional file 1: Figure S1.
*Sc*AGU115 codon optimized sequence used for the expression in Arabidopsis. (A) Comparison of native and codon-optimized *Sc*AGU115 sequences using CLUSTAL 2.1 alignment. (B) Nucleotide sequence corresponding to the native *Sc*AGU115 signal peptide and the signal peptide originating from hybrid aspen cellulase *Ptt*CEL9B3 used in the expression vector. Additional file 2: Figure S2. Screening of Arabidopsis homozygotic lines expressing *Sc*AGU115 α-glucuronidase. (A) Gene expression analysis and amplification of *Actin2* gene was used as the internal control. (B) Soluble protein was extracted from stem tissues of the 7 weeks old plants, and was analyzed for the α-glucuronidase activity using Megazyme kit. The error bar represents the standard deviation of three biological replicates. Additional file 3: Figure S3. Relative content of substituted and non-substituted internal Xyl*p* residues in the *O*-acetylglucuronoxylan of transgenic (lines 4, 5) and WT plants based on integration of cross peaks in 2D qHSQC spectra. *X*2, [2-*O*-Ac]-β-D-Xyl*p*; X3, [3-*O*-Ac]-β-D-Xyl*p*; X23, [2,3-*O*-Ac]-β-D-Xyl*p*; X3G2, [α-D-(Me)Glc*p*A*(1 → 2)][3-O-Ac]-β-D-Xyl*p*; XG2, [α-D-(Me)Glc*p*A*(1 → 2)]-β-D-Xyl*p*; * representing both Glc*p*A and MeGlc*p*A since their cross peak signals were unresolved in the spectra. The error bar represents the standard deviation of two biological replicates. Additional file 4: Table S1. Comparison of carbohydrate and lignin components in the stem tissues. The guaiacyl, syringyl and *p*-hydroxyphenyl lignins; and carbohydrate fraction in the transgenic (lines 4, 5) and WT plants were analyzed by pyrolysis-gas chromatography/ mass spectrometry. The ± represents the standard deviation of five biological replicates. **p* value < 0.05, *t*-test. Additional file 5: Figure S4. Digestibility of the main stems from transgenic (lines 4, 5) and WT plants. The main stem tissues were ground and pre-treated by (A) water, and (B) 0.5M NaOH before the enzymatic hydrolysis. Error bar represents standard deviation of three biological replicates.

## Abbreviations

AcGX: *O*-acetylglucuronoxylans
bp: Base pair
BSA: Bovine serum albumin
Col-0: Columbia ecotype
DMSO: Dimethylsulfoxide
EGTA: Ethylene glycol-bis(2-aminoethylether)-N,N,N’,N’-tetraacetic acid
GFP: Green fluorescence protein
IGEPAL: Octylphenyl-polyethylene glycol
GC: Gas chromatography
GlcAT: Glucuronyltransferase
GUX: GlucUronic acid substitution of Xylan
HSQC: Heteronuclear single quantum coherence spectroscopy
i.d.: Internal diameter
MBTH: 3-methyl-2benzothiazolinonehydrazone
MES: 2-(N-morpholino)ethanesulfonic acid
MWCO: Molecular weight cut-off
NaOAc: Sodium acetate
NMR: Nuclear magnetic resonance
PIPES: 1,4-piperazinediethanesulfonic acid
PVDF: Polyvinylidene fluoride
PVPP: Polyvinylpolypyrrolidone
Py-GC/MS: Pyrolysis-GC/mass spectrometry
SDS-PAGE: Sodium dodecyl sulphate-polyacrylamide gel electrophoresis
TLC: Thin layer chromatography
XUXX: β-D-Xylp-(1 → 4)-[4-O-methyl-α-D-GlcpA-(1 → 2)]-β-D-Xylp-(1 → 4)- β-D-Xylp-(1 → 4)-D-Xyl
WT: Wild type

## References

[1] Himmel ME, Ding S, Johnson DK, Adney WS, Nimlos MR, Brady JW (2007). Biomass recalcitrance: engineering plants and enzymes for biofuels production. Science.

[2] Pauly M, Keegstra K (2008). Cell-wall carbohydrates and their modification as a resource for biofuels. Plant J.

[3] Mellerowicz EJ, Gorshkova TA (2011). Tensional stress generation in gelatinous fibres: a review and possible mechanism based on cell-wall structure and composition. J Exp Botany.

[4] Carpita NC, Gibeaut DM (1993). Structural models of primary cell walls in flowering plants: consistency of molecular structure with the physical properties of the walls during growth. Plant J.

[5] Cosgrove DJ (2005). Growth of the plant cell wall. Nat Rev Mol Cell Biol.

[6] Scheller HV, Ulvskov P (2010). Hemicelluloses. Annu Rev Plant Biol.

[7] Ebringerová A, Heinze T (2000). Xylan and xylan derivatives -- biopolymers with valuable properties, 1. Naturally occurring xylans structures, isolation procedures and properties. Macromol Rapid Commun.

[8] Cheng K, Sorek H, Zimmermann H, Wemmer DE, Pauly M (2013). Solution-state 2D NMR spectroscopy of plant cell walls enabled by a dimethylsulfoxide-d6/1-ethyl-3-methylimidazolium acetate solvent. Anal Chem.

[9] Chong SL, Virkki L, Maaheimo H, Juvonen M, Derba-Maceluch M, Koutaniemi S (2014). *O*-Acetylation of glucuronoxylan in *Arabidopsis thaliana* wild type and its change in xylan biosynthesis mutants. Glycobiology.

[10] Evtuguin D, Tomás J, Silva AS, Neto C (2003). Characterization of an acetylated heteroxylan from *Eucalyptus globulus* Labill. Carbohydr Res.

[11] Naran R, Black S, Decker SR, Azadi P (2009). Extraction and characterization of native heteroxylans from delignified corn stover and aspen. Cellulose.

[12] Teleman A, Tenkanen M, Jacobs A, Dahlman O (2002). Characterization of *O*-acetyl-(4-*O*-methylglucurono)xylan isolated from birch and beech. Carbohydr Res.

[13] Yuan Y, Teng Q, Zhong R, Ye Z (2013). The Arabidopsis DUF231 domain-containing protein ESK1 mediates 2-*O*- and 3-*O*-acetylation of xylosyl residues in xylan. Plant and Cell Physiol.

[14] Shatalov AA, Evtuguin DV, Pascoal Neto C (1999). (2-*O*-α-D-Galactopyranosyl-4-*O*-methyl-α-D-glucurono)-D-xylan from *Eucalyptus globulus* Labill. Carbohydr Res.

[15] Balakshin MY, Capanema EA, Chang H (2007). MWL fraction with a high concentration of lignin-carbohydrate linkages: isolation and 2D NMR spectroscopic analysis. Holzforschung.

[16] Takahashi N, Koshijima T (1988). Ester linkages between lignin and glucuronoxylan in a lignin-carbohydrate complex from beech (*Fagus crenata*) wood. Wood Sci Technol.

[17] Brown DM, Zhang Z, Stephens E, Dupree P, Turner SR (2009). Characterization of IRX10 and IRX10-like reveals an essential role in glucuronoxylan biosynthesis in Arabidopsis. Plant J.

[18] Wu A, Rihouey C, Seveno M, Hoernblad E, Singh SK, Matsunaga T (2009). The Arabidopsis IRX10 and IRX10-LIKE glycosyltransferases are critical for glucuronoxylan biosynthesis during secondary cell wall formation. Plant J.

[19] Wu A, Hörnblad E, Voxeur A, Gerber L, Rihouey C, Lerouge P (2010). Analysis of the Arabidopsis IRX9/IRX9-L and IRX14/IRX14-L pairs of glycosyltransferase genes reveals critical contributions to biosynthesis of the hemicellulose glucuronoxylan. Plant Physiol.

[20] Lee C, Zhong R, Richardson EA, Himmelsbach DS, McPhail BT, Ye Z (2007). The *PARVUS* gene is expressed in cells undergoing secondary wall thickening and is essential for glucuronoxylan biosynthesis. Plant Cell Physiol.

[21] Peña MJ, Zhong R, Zhou G, Richardson EA, O'Neill MA, Darvill AG (2007). Arabidopsis *irregular xylem8* and *irregular xylem9*: Implications for the complexity of glucuronoxylan biosynthesis. Plant Cell.

[22] Zhong R, Pena MJ, Zhou G, Nairn CJ, Wood-Jones A, Richardson EA (2005). Arabidopsis *fragile fiber8*, which encodes a putative glucuronyltransferase, is essential for normal secondary wall synthesis. Plant Cell.

[23] Mortimer JC, Miles GP, Brown DM, Zhang Z, Segura MP, Weimar T (2010). Absence of branches from xylan in Arabidopsis *gux* mutants reveals potential for simplification of lignocellulosic biomass. Proc Natl Acad Sci U S A.

[24] Lee C, Teng Q, Zhong R, Ye Z (2012). Arabidopsis GUX proteins are glucuronyltransferases responsible for the addition of glucuronic acid side chains onto xylan. Plant Cell Physiol.

[25] Lee C, Teng Q, Zhong R, Ye Z (2011). The four Arabidopsis *REDUCED WALL ACETYLATION* genes are expressed in secondary wall-containing cells and required for the acetylation of xylan. Plant Cell Physiol.

[26] Manabe Y, Verhertbruggen Y, Gille S, Harholt J, Chong S, Pawar PM (2013). RWA proteins play vital and distinct roles in cell wall *O*-acetylation in *Arabidopsis thaliana*. Plant Physiol.

[27] Bae H, Kim HJ, Kim YS (2008). Production of a recombinant xylanase in plants and its potential for pulp biobleaching applications. Bioresour Technol.

[28] Borkhardt B, Harholt J, Ulvskov P, Ahring BK, Jørgensen B, Brinch-Pedersen H (2010). Autohydrolysis of plant xylans by apoplastic expression of thermophilic bacterial endo-xylanases. Plant Biotechnol.

[29] Buanafina M, Langdon T, Dalton S, Morris P (2012). Expression of a *Trichoderma reesei* β-1,4 endo-xylanase in tall fescue modifies cell wall structure and digestibility and elicits pathogen defence responses. Planta.

[30] Herbers K, Wilke I, Sonnewald U (1995). A thermostable xylanase from *Clostridium thermocellum* expressed at high levels in the apoplast of transgenic tobacco has no detrimental effects and is easily purified. Nat Biotech.

[31] Kimura T, Mizutani T, Tanaka T, Koyama T, Sakka K, Ohmiya K (2003). Molecular breeding of transgenic rice expressing a xylanase domain of the *xynA* gene from *Clostridium thermocellum*. Appl Microbiol Biotechnol.

[32] Weng X, Huang Y, Hou C, Jiang D (2013). Effects of an exogenous xylanase gene expression on the growth of transgenic rice and the expression level of endogenous xylanase inhibitor gene RIXI. J Sci Food Agric.

[33] Pogorelko G, Fursova O, Lin M, Pyle E, Jass J, Zabotina O (2011). Post-synthetic modification of plant cell walls by expression of microbial hydrolases in the apoplast. Plant Mol Biol.

[34] Pogorelko G, Lionetti V, Fursova O, Sundaram RM, Qi M, Whitham SA (2013). Arabidopsis and *Brachypodium distachyon* transgenic plants expressing *Aspergillus nidulans* acetylesterases have decreased degree of polysaccharide acetylation and increased resistance to pathogens. Plant Physiol.

[35] Tsai AY, Canam T, Gorzsás A, Mellerowicz EJ, Campbell MM, Master ER (2012). Constitutive expression of a fungal glucuronoyl esterase in Arabidopsis reveals altered cell wall composition and structure. Plant Biotechnol.

[36] Latha Gandla M, Derba-Maceluch M, Liu X, Gerber L, Master ER, Mellerowicz EJ (2015). Expression of a fungal glucuronoyl esterase in Populus: Effects on wood properties and saccharification efficiency. Phytochemistry.

[37] de Vries RP, Poulsen CH, Madrid S, Visser J (1998). *aguA*, the gene encoding an extracellular alpha-glucuronidase from *Aspergillus tubingensis*, is specifically induced on xylose and not on glucuronic acid. J Bacteriol.

[38] de Vries R, Battaglia E, Coutinho P, Henrissat B, Visser J, Hofrichter M (2011). (Hemi-)cellulose degrading enzymes and their encoding genes from aspergillus and trichoderma. The Mycota: A comprehensive treatise on fungi as experimental systems for basic and applied research.

[39] Siika-aho M, Tenkanen M, Buchert J, Puls J, Viikari L (1994). An α-glucuronidase from *Trichoderma reesei* Rut C-30. Enzyme Microb Technol.

[40] Tenkanen M, Siika-aho M (2000). An α-glucuronidase of *Schizophyllum commune* acting on polymeric xylan. J Biotechnol.

[41] Chong SL, Battaglia E, Coutinho P, Henrissat B, Tenkanen M, Vries R (2011). The α-glucuronidase Agu1 from *Schizophyllum commune* is a member of a novel glycoside hydrolase family (GH115). Appl Microbiol Biotechnol.

[42] Burgess-Brown NA, Sharma S, Sobott F, Loenarz C, Oppermann U, Gileadi O (2008). Codon optimization can improve expression of human genes in *Escherichia coli*: A multi-gene study. Protein Expr Purif.

[43] Rudsander U, Denman S, Raza S, Teeril TT (2003). Molecular features of family GH9 cellulases in hybrid Aspen and the filamentous fungus *Phanerochaete chrysosporium*. J Appl Glycosci.

[44] Takahashi J, Rudsander UJ, Hedenström M, Banasiak A, Harholt J, Amelot N (2009). KORRIGAN1 and its Aspen homolog *Ptt*Cel9A1 decrease cellulose crystallinity in Arabidopsis stems. Plant Cell Physiol.

[45] Campbell TN, Choy FYM (2001). The effect of pH on green fluorescent protein: a brief review. Mol Biol Today.

[46] Zimmer M (2002). Green Fluorescent Protein (GFP): Applications, structure, and related photophysical behavior. Chem Rev.

[47] Minic Z, Jouanin L (2006). Plant glycoside hydrolases involved in cell wall polysaccharide degradation. Plant Physiol Bioch.

[48] Wolf S, Hématy K, Höfte H (2012). Growth control and cell wall signaling in plants. Annu Rev Plant Biol.

[49] Zipfel C (2009). Early molecular events in PAMP-triggered immunity. Curr Opin Plant Biol.

[50] Lange BM, Lapierre C, Sandermann H (1995). Elicitor-induced spruce stress lignin (structural similarity to early developmental lignins). Plant Physiol.

[51] Koutaniemi S, Guillon F, Tranquet O, Bouchet B, Tuomainen P, Virkki L (2012). Substituent-specific antibody against glucuronoxylan reveals close association of glucuronic acid and acetyl substituents and distinct labeling patterns in tree species. Planta.

[52] Guillon F, Tranquet O, Quillien L, Utille J, Ordaz Ortiz JJ, Saulnier L (2004). Generation of polyclonal and monoclonal antibodies against arabinoxylans and their use for immunocytochemical location of arabinoxylans in cell walls of endosperm of wheat. J Cereal Sci.

[53] Harper AD, Bar-Peled M (2002). Biosynthesis of UDP-Xylose. Cloning and characterization of a novel Arabidopsis gene family, *UXS*, encoding soluble and putative membrane-bound UDP-glucuronic acid decarboxylase isoforms. Plant Physiol.

[54] Busse-Wicher M, Gomes TCF, Tryfona T, Nikolovski N, Stott K, Grantham NJ (2014). The pattern of xylan acetylation suggests xylan may interact with cellulose microfibrils as a two-fold helical screw in the secondary plant cell wall of *Arabidopsis thaliana*. Plant J.

[55] McKee LS, Peña MJ, Rogowski A, Jackson A, Lewis RJ, York WS (2012). Introducing endo-xylanase activity into an exo-acting arabinofuranosidase that targets side chains. Proc Natl Acad Sci U S A.

[56] Kolenova K, Ryabova O, Vrsanska M, Biely P (2010). Inverting character of family GH115 α-glucuronidases. FEBS Lett.

[57] Rogowski A, Baslé A, Farinas CS, Solovyova A, Mortimer JC, Dupree P (2014). Evidence that GH115 α-glucuronidase activity, which is required to degrade plant biomass, is dependent on conformational flexibility. J Biol Chem.

[58] Karimi M, Inzé D, Depicker A (2002). GATEWAY™ vectors for Agrobacterium-mediated plant transformation. Trends Plant Sci.

[59] Clough SJ, Bent AF (1998). Floral dip: a simplified method forAgrobacterium-mediated transformation of *Arabidopsis thaliana*. Plant J.

[60] Franková L, Fry SC (2011). Phylogenetic variation in glycosidases and glycanases acting on plant cell wall polysaccharides, and the detection of transglycosidase and trans-β-xylanase activities. Plant J.

[61] Sambrook JF, Russell DW. Molecular cloning: a laboratory manual*.* 3rd ed. New York: Cold Spring Harbor Laboratory Press; 2001.

[62] Chong SL, Nissila T, Ketola RA, Koutaniemi S, Derba-Maceluch M, Mellerowicz EJ (2011). Feasibility of using atmospheric pressure matrix-assisted laser desorption/ionization with ion trap mass spectrometry in the analysis of acetylated xylooligosaccharides derived from hardwoods and *Arabidopsis thaliana*. Anal Bioanal Chem.

[63] Sundberg A, Sundberg K, Lillandt C, Holmbom B (1996). Determination of hemicelluloses and pectins in wood and pulp fibers by acid methanolysis and gas chromatography. Nord Pulp Pap Res J.

[64] Chong SL, Koutaniemi S, Virkki L, Pynnönen H, Tuomainen P, Tenkanen M (2013). Quantitation of 4-*O*-methylglucuronic acid from plant cell walls. Carbohydr Polym.

[65] Gerber L, Eliasson M, Trygg J, Moritz T, Sundberg B (2012). Multivariate curve resolution provides a high-throughput data processing pipeline for pyrolysis-gas chromatography/mass spectrometry. J Anal Appl Pyrolysis.

[66] Gomez L, Whitehead C, Barakate A, Halpin C, McQueen-Mason S (2010). Automated saccharification assay for determination of digestibility in plant materials. Biotechnol for Biofuels.

