# Supplementary material for: Active fungal GH115 α-glucuronidase produced in Arabidopsis thaliana affects only the UX1-reactive glucuronate decorations on native glucuronoxylans
Source: BMC Biotechnol. 2015 Jun 18;15:56. doi: 10.1186/s12896-015-0154-8 (PMC4472178; doi:10.1186/s12896-015-0154-8)
Supplement: Additional file 1: Figure S1. — ScAGU115 codon optimized sequence used for the expression in Arabidopsis. (A) Comparison of native and codon-optimized ScAGU115 sequences using CLUSTAL 2.1 alignment. (B) Nucleotide sequence corresponding to the native ScAGU115 signal peptide and the signal peptide originating from hybrid aspen cellulase PttCEL9B3 used in the expression vector. [file 12896_2015_154_MOESM1_ESM.docx]

**A**

CLUSTAL 2.1 multiple sequence alignment

ScAGU115optimized CTTTCAGAGGGTAATTGTGTGTCTTTTGATGCTTCATCTGGTGGATTCCCACTCGTTGGC

ScAGU115 TTGAGCGAGGGCAACTGCGTCTCTTTCGACGCGTCCTCCGGCGGCTTCCCTCTCGTCGGG

* : .***** ** ** ** ***** ** ** **.** ** **.*****:***** **

ScAGU115optimized GCAACCATTATAACATCAGAGGATGATTTTCCTGGTGTGCATAGGGCTGTTGCAGATTTC

ScAGU115 GCGACCATCATCACCTCCGAGGACGACTTCCCGGGCGTGCATCGCGCCGTGGCCGACTTC

**.***** **.**.**.***** ** ** ** ** ******.* ** ** **.** ***

ScAGU115optimized ATAGTGGATGTTGCTAACGTTACCGGGACAGCACCAGCTGCTTTTAATTTGACAGATTTG

ScAGU115 ATCGTCGACGTCGCGAACGTGACCGGTACCGCCCCCGCCGCCTTCAACCTCACAGACCTC

**.** ** ** ** ***** ***** **.**.**.** ** ** ** * ***** *

ScAGU115optimized GAAACATTGCCATCATCAGCTCTCATCGTGGGTAGCGTTAACTCCACAATCATTTCACAA

ScAGU115 GAGACACTCCCCTCCTCCGCCCTCATCGTCGGCAGCGTCAACTCCACCATCATCTCCCAG

**.*** * **.**.**.** ******** ** ***** ********.***** **.**.

ScAGU115optimized TTGTCAAATTCTTATGCTGTTGATTTGTCTGCACTTACTGGACAGTGGGAGAAGTTTGCA

ScAGU115 CTTTCGAACTCGTACGCCGTCGACCTTAGCGCGCTCACGGGCCAGTGGGAGAAGTTCGCC

* **.** ** ** ** ** ** * : **.** ** **.************** **.

ScAGU115optimized AGTGGACCAGCTCCTGATGCATTGCCAGGGGTGAAGAATGCTTATGTTATAGCAGGATCA

ScAGU115 AGCGGGCCGGCGCCCGACGCGCTCCCTGGCGTCAAGAACGCGTACGTTATCGCTGGGTCG

** **.**.** ** ** **. * **:** ** ***** ** ** *****.**:**.**.

ScAGU115optimized GATAAAAGAGGTACAATCTTCGGAGTGTATGAACTTAGTGAGCAAATTGGGGTTAGCCCT

ScAGU115 GACAAACGTGGTACAATCTTCGGCGTCTACGAGCTTTCTGAACAGATCGGCGTTTCCCCG

** ***.*:**************.** ** **.***: ***.**.** ** ***: ***

ScAGU115optimized TGGTACTGGTGGGCTGATGTGCCTATATCAAAGCATGAAGAGGTTTATTTGCTTCCATGT

ScAGU115 TGGTACTGGTGGGCCGACGTTCCCATTTCGAAGCACGAGGAGGTCTACCTGCTCCCCTGC

************** ** ** ** **:**.***** **.***** ** **** **.**

ScAGU115optimized GCACAGGGCCCACCTACTGTTAAATACAGGGGTATTTTTCTTAACGATGAAGAGCCTGCT

ScAGU115 GCGCAAGGGCCACCGACGGTGAAGTACCGCGGCATATTCCTCAACGACGAGGAGCCCGCG

**.**.** ***** ** ** **.***.* ** **:** ** ***** **.***** **

ScAGU115optimized CTCTCAAACTGGGCTAAGGCAAAATTCACTAATGGTACATTGTCAGAATTAATGCATAGC

ScAGU115 CTATCGAACTGGGCCAAGGCAAAGTTCACGAATGGCACGCTTTCGGAGCTGATGCACTCG

**.**.******** ********.***** ***** **. * **.**. *.***** :

ScAGU115optimized CCTTTTAACCATCACTTCTACGAAAAGCTCTTCGAATTGATTTTGAGATTGAAGGCTAAC

ScAGU115 CCGTTCAACCATCACTTCTATGAGAAGCTATTCGAGCTTATTCTTCGGCTGAAGGCGAAT

** ** ************** **.*****.*****. * *** * .*. ******* **

ScAGU115optimized CACTTATGGCCAGCTATGTGGGGTTCTGCATTCTGCGTTGATGATGAGTTGAATCAACCT

ScAGU115 CATCTGTGGCCAGCCATGTGGGGCAGCGCATTCTGTGTCGACGATGAGCTCAACCAGCCG

** *.******** ******** : ******** ** ** ****** * ** **.**

ScAGU115optimized ATGGCAGATTATTACGGGGTTGTGATGGGCACTTCACACCAGGAACCAATGATGAGGTCT

ScAGU115 ATGGCGGATTACTACGGCGTGGTGATGGGGACCAGCCACCAAGAACCCATGATGCGCTCG

*****.***** ***** ** ******** ** : .*****.*****.******.* **

ScAGU115optimized ACCCCTAATGAGTGGAACTTGTTTGGTAATGGTACATGGGATTATTCTACCAATGCAGAT

ScAGU115 ACGCCGAACGAGTGGAACCTCTTCGGTAACGGCACCTGGGATTACAGCACGAACGCGGAC

** ** ** ********* * ** ***** ** **.******** : ** ** **.**

ScAGU115optimized AACATCTACCCATATCTTTACGAAGGTGCTAAAAGGGCAAGTCCTTATGAGTCAATTTTC

ScAGU115 AACATCTATCCCTACCTCTACGAAGGTGCTAAGCGGGCGTCCCCGTACGAGTCGATCTTC

******** **.** ** **************..****.: ** ** *****.** ***

ScAGU115optimized TCTATGGGAATGAGAGGGGCTGGCGATCTTCCACTCTCAGATGAAACTAACATCGAATTG

ScAGU115 AGCATGGGTATGCGCGGGGCGGGTGATTTACCCCTCTCAGACGAGACGAACATCGAACTG

: *****:***.*.***** ** *** *:**.******** **.** ********* **

ScAGU115optimized CTCACCCAAATAGTTGCAGATGAAAGGCAGATTTTGTCCGATGTGTTTAATACATCAGAT

ScAGU115 CTCACGCAGATCGTCGCGGACGAGCGTCAGATTCTGAGCGATGTCTTCAACACGTCGGAC

***** **.**.** **.** **..* ****** **: ****** ** ** **.**.**

ScAGU115optimized GTTACAACTATACCACAAATATGGACTCTTTTCCAAGAAGTGCAGGGGTATTACGAGCAG

ScAGU115 GTGACGACTATTCCGCAGATATGGACTTTGTTCCAGGAGGTCCAGGGCTACTACGAACAG

** **.*****:**.**.********* * *****.**.** ***** ** *****.***

ScAGU115optimized GGCATGGAAGTTCCTGAGGATATTACATTGCTTTGGTCAGATGATAACTGGGGAAACGTT

ScAGU115 GGTATGGAAGTTCCGGAGGATATCACGCTGCTTTGGTCGGATGATAACTGGGGCAATGTC

** *********** ******** **. **********.**************.** **

ScAGU115optimized AGGAGATTTCCAACTCCTAATGAGAGGAACAGAACCGGGGGCGCTGGAGTTTATTACCAT

ScAGU115 CGTCGTTTCCCTACGCCAAACGAGAGGAACAGGACTGGTGGCGCCGGTGTCTACTACCAC

.* .*:** **:** **:** ***********.** ** ***** **:** ** *****

ScAGU115optimized GTGGATTTCGTTGGAGATCCAAGGGATTATAAGTGGATTGAATCCTCACAAATTTCAAAG

ScAGU115 GTAGACTTCGTCGGAGACCCGCGCGACTACAAGTGGATCGAGAGCTCCCAAATCTCCAAG

**.** ***** ***** **..* ** ** ******** **.: ***.***** **.***

ScAGU115optimized ATATATGAGCAGATGAGTTTGGCTGTTGAATACGAGGCAACAAGGGTGTGGATGTTAAAT

ScAGU115 ATCTACGAGCAGATGTCCCTCGCCGTCGAGTACGAGGCCACCCGCGTCTGGATGCTCAAT

**.** *********: * ** ** **.********.**..* ** ****** *.***

ScAGU115optimized GTTGGAGATTTGAAGCCTTATGAAAGATCAACAGAGTTTTTCTTAACTTTGGGATGGAAC

ScAGU115 GTCGGCGACCTCAAGCCCTACGAGCGCTCCACTGAGTTCTTCCTCACCCTCGGCTGGAAC

** **.** * ***** ** **..*.**.**:***** *** *.** * **.******

ScAGU115optimized GCTTCAAGGTGGACTCCTGATAATGTGGGGACATTTGTTACTTCTTGGGCAAAAAGGGAT

ScAGU115 GCCAGCCGCTGGACACCCGACAACGTCGGCACCTTCGTCACCAGCTGGGCGAAGCGTGAC

** : ..* *****:** ** ** ** ** **.** ** ** : *****.**..* **

ScAGU115optimized TTCCAAGTTGAAGATGCTGAAGCAGAGGCTATAGCAGAGATAATCGCTAACTTCACAAAG

ScAGU115 TTCCAGGTCGAGGACGCGGAGGCGGAGGCCATTGCAGAGATCATCGCCAACTTCACGAAG

*****.** **.** ** **.**.***** **:********.***** ********.***

ScAGU115optimized TTGAACGCAAGGAGAAAACCAGAACTCTGGAACAGCACCACATATTCCTTAACTAATTAC

ScAGU115 CTGAACGCGCGGAGGAAGCCTGAGTTGTGGAACAGCACGACGTACAGCCTGACGAACTAT

*******..****.**.**:**. * *********** **.** : * *.** ** **

ScAGU115optimized AGGGAAGCTCAGACTACCCTTGATGAGTGGAATGCTATCAGAAACACTAGTACCGCAATC

ScAGU115 CGGGAGGCGCAGACGACTTTGGACGAGTGGAACGCCATTCGGAATACCTCGACGGCGATC

.****.** ***** ** * ** ******** ** ** .*.** ** : ** **.***

ScAGU115optimized TATGATGGTTTGCCATCAGATGTTCAACCTGCTTACTTTCAGCTTGTGCATCACGCAGTT

ScAGU115 TACGACGGACTCCCGTCCGACGTCCAGCCCGCGTACTTCCAGCTCGTGCATCATGCCGTG

** ** **: * **.**.** ** **.** ** ***** ***** ******** **.**

ScAGU115optimized CTTGCTTCAGCAAACCTCGGGGAAATGCTTATTCTCGCTGGCTTGAATAACCTTTATGCT

ScAGU115 CTCGCCAGCGCCAACCTCGGCGAGATGCTGATTTTGGCCGGGCTCAACAACCTCTATGCA

** ** : .**.******** **.***** *** * ** ** * ** ***** *****:

ScAGU115optimized TCCCAAGCAAGGTTATCAACAAATGATTTGGCAGATAAGGTTCAGGAATTATTCGAGTAC

ScAGU115 TCGCAGGCTCGCCTCAGCACGAACGACCTTGCGGATAAGGTGCAGGAGCTATTTGAGTAT

** **.**:.* *.: .**.** ** * **.******** *****. **** *****

ScAGU115optimized GATTACGATTTGGAAGTTGAGTATCATTCTATTTTGGATGGAAAGTGGAATCACATGATG

ScAGU115 GATTACGATCTCGAGGTCGAATACCATAGCATCCTCGACGGAAAATGGAACCATATGATG

********* * **.** **.** ***: ** * ** *****.***** ** ******

ScAGU115optimized GATCAAACTCACGTTGGGTATTACTATTGGCAACAGCCAATGACAAATACTATGCCTGCT

ScAGU115 GATCAGACCCACGTCGGATACTACTATTGGCAGCAACCTATGACCAACACTATGCCTGCC

*****.** ***** **.** ***********.**.**:*****.** ***********

ScAGU115optimized GTGAACAGGGTTCAAAGTAGGAGACAGGCTCTTGCAGGGGTTATGAGAATTATACCTGAA

ScAGU115 GTTAACCGCGTTCAGTCGAGAAGGCAAGCTCTTGCCGGTGTCATGCGAATCATCCCCGAG

** ***.* *****.: **.**.**.********.** ** ***.**** **.** **.

ScAGU115optimized GGGACAGCTGGCGCATGGCCAGGAGATAATCCTTATCAGTGTGCAAAGGGTTACGATTGC

ScAGU115 GGTACAGCCGGTGCTTGGCCCGGCGACAACCCATACCAGTGCGCGAAAGGCTACGACTGT

** ***** ** **:*****.**.** ** **:** ***** **.**.** ***** **

ScAGU115optimized GGAGATCCAACTATCTATATTGATAGGTACAGTCCTATTAGCGATAGATATGTGGATGTT

ScAGU115 GGTGACCCCACCATCTACATCGACCGCTACTCTCCCATCTCCGACCGCTACGTCGACGTC

**:** **.** ***** ** ** .* ***: *** ** : *** .*.** ** ** **

ScAGU115optimized AGTGCTGGTGGACCAGCACCTTTTAGCTGGAATGTGACATCCAACGCTTCATGGGTTAGT

ScAGU115 TCCGCCGGTGGTCCTGCACCCTTCTCCTGGAACGTCACCAGCAATGCGAGCTGGGTGTCG

: ** *****:**:***** ** : ****** ** **.: *** ** : .***** :

ScAGU115optimized ACTAGCTTGCAAGGGGGCGATATATCTCCAGATAGTAAGGAAGCTAGACTCTATTTGTCT

ScAGU115 ACGTCCCTTCAAGGCGGCGACATATCTCCTGACAGCAAGGAGGCGCGGTTGTATCTCTCT

** : * * ***** ***** ********:** ** *****.** .*. * *** * ***

ScAGU115optimized GTGAACGATTGGAGTGCAGTTAACGGAACATCTTACGCTCAGTTGACTTTTGTTGCAATA

ScAGU115 GTCAACGATTGGAGTGCTGTGAACGGAACAAGCTATGCGCAGCTGACCTTCGTCGCGATC

** **************:** *********: ** ** *** **** ** ** **.**.

ScAGU115optimized TCTAGTATCGATGATCCTCTTACCGTGAATCTTACACTCGTTGCTTATAACCCAGGTGCT

ScAGU115 TCGAGCATCGACGATCCGCTGACGGTCAACCTCACGCTGGTTGCTTACAACCCGGGTGCT

** ** ***** ***** ** ** ** ** ** **.** ******** *****.******

ScAGU115optimized CCTGCAGAAGGTTTTACAGGATACGTTGAGGGAGATGGAGTTGTGTCATTCGAAGCATCT

ScAGU115 CCGGCTGAGGGCTTCACCGGCTACGTTGAGGGTGATGGTGTGGTGTCCTTCGAGGCCTCG

** **:**.** ** **.**.***********:*****:** *****.*****.**.**

ScAGU115optimized CACGCTGCAAGGAATACCCCAGTGGATGATCTCGCTTGGACAGTTATTCCTGATTATGGT

ScAGU115 CATGCAGCGAGGAATACGCCCGTTGACGATCTCGCGTGGACCGTAATTCCCGATTATGGG

** **:**.******** **.** ** ******** *****.**:***** ********

ScAGU115optimized AGGACCTTAGCAGGAGTTACACCTTGGCCAAGGGGAGATAAGAATTTTACCGCTGGGACA

ScAGU115 CGCACATTGGCAGGCGTCACTCCCTGGCCGCGCGGCGACAAGAACTTCACTGCCGGTACC

.* **.**.*****.** **:** *****..* **.** ***** ** ** ** ** **.

ScAGU115optimized GGCCCATCTTTGGAGTACGATTTCTACACATTCGGAAATGTTAGCTCCGCTAACGTGACT

ScAGU115 GGTCCGTCTTTGGAGTATGACTTCTACACCTTCGGCAACGTCAGCAGCGCCAACGTCACC

** **.*********** ** ********.*****.** ** ***: *** ***** **

ScAGU115optimized GTTTATCTTTCCCCTTCATGGAATGCACAGGGTATGAATAACTCTCTCAGTTACGGGTTA

ScAGU115 GTCTACCTGTCGCCTTCCTGGAACGCGCAAGGCATGAATAACTCCCTGTCGTACGGCCTG

** ** ** ** *****.***** **.**.** *********** ** : ***** *.

ScAGU115optimized CAGGTTGATGAATCAGATATTGATGTTGTGGAGTATATTCCATACCCTACAACTATAGGT

ScAGU115 CAAGTAGACGAGTCCGACATCGACGTCGTCGAGTACATCCCATACCCGACGACGATCGGG

**.**:** **.**.** ** ** ** ** ***** ** ******** **.** **.**

ScAGU115optimized GGAAAGCCAGCTGAATGGGCAGGGTTGGATGGCTTTGTTGCTAATAACATAGCAACCGGT

ScAGU115 GGCAAGCCCGCGGAGTGGGCCGGCTTGGACGGCTTCGTGGCGAACAACATCGCCACGGGC

**.*****.** **.*****.** ***** ***** ** ** ** *****.**.** **

ScAGU115optimized AAATCAAACTTCACACTTTCCGGTGCTGGAGCACATACTTTAAAGTTGTGGATGACTCAA

ScAGU115 AAGAGCAACTTCACGCTGAGTGGTGCTGGGGCACATACGCTCAAGCTCTGGATGACGCAG

**.: .********.** : ********.******** *.*** * ******** **.

ScAGU115optimized CCTACCGTTGTGGTTCAGAAAATAGTTATCGATTTGGGGGGCGCTCTTGCAAGCTATCTC

ScAGU115 CCAACCGTGGTGGTGCAGAAGATCGTGATAGATCTTGGCGGCGCACTCGCAAGCTACCTC

**:***** ***** *****.**.** **.*** * ** *****:** ******** ***

ScAGU115optimized GGCCCACCTGAGTCCGTGCACGTTGGTGCTTGA

ScAGU115 GGGCCTCCCGAGAGCGTGCACGTGGGAGCTTGA

** **:** ***: ********* **:******

**B**

> Signal Peptide *Ptt*Cel9B3

ATGAGAAGGGGAGCTTCTTTCTGCCTCTTGTTTTCCCTGTCACTTGTTCTCTTGGGTTTTGTCCAAGCC

>Signal Peptide *Sc*AGU115

ATGTTCAGTCGCGCGGCCCTTGTGGCGGCGGCCCTGGCTGCTCAGCGCGTCTATGCC

**Fig. S1. *Sc*AGU115 codon optimized sequence used for the expression in Arabidopsis.** (A) Comparison of native and codon-optimized *Sc*AGU115 sequences using CLUSTAL 2.1 alignment. (B) Nucleotide sequence corresponding to the native *Sc*AGU115 signal peptide and the signal peptide originating from hybrid aspen cellulase *Ptt*CEL9B3 used in the expression vector.
