# Supplementary figures and images for: Active fungal GH115 α-glucuronidase produced in Arabidopsis thaliana affects only the UX1-reactive glucuronate decorations on native glucuronoxylans
Source: BMC Biotechnol. 2015 Jun 18;15:56. doi: 10.1186/s12896-015-0154-8 (PMC4472178; doi:10.1186/s12896-015-0154-8)

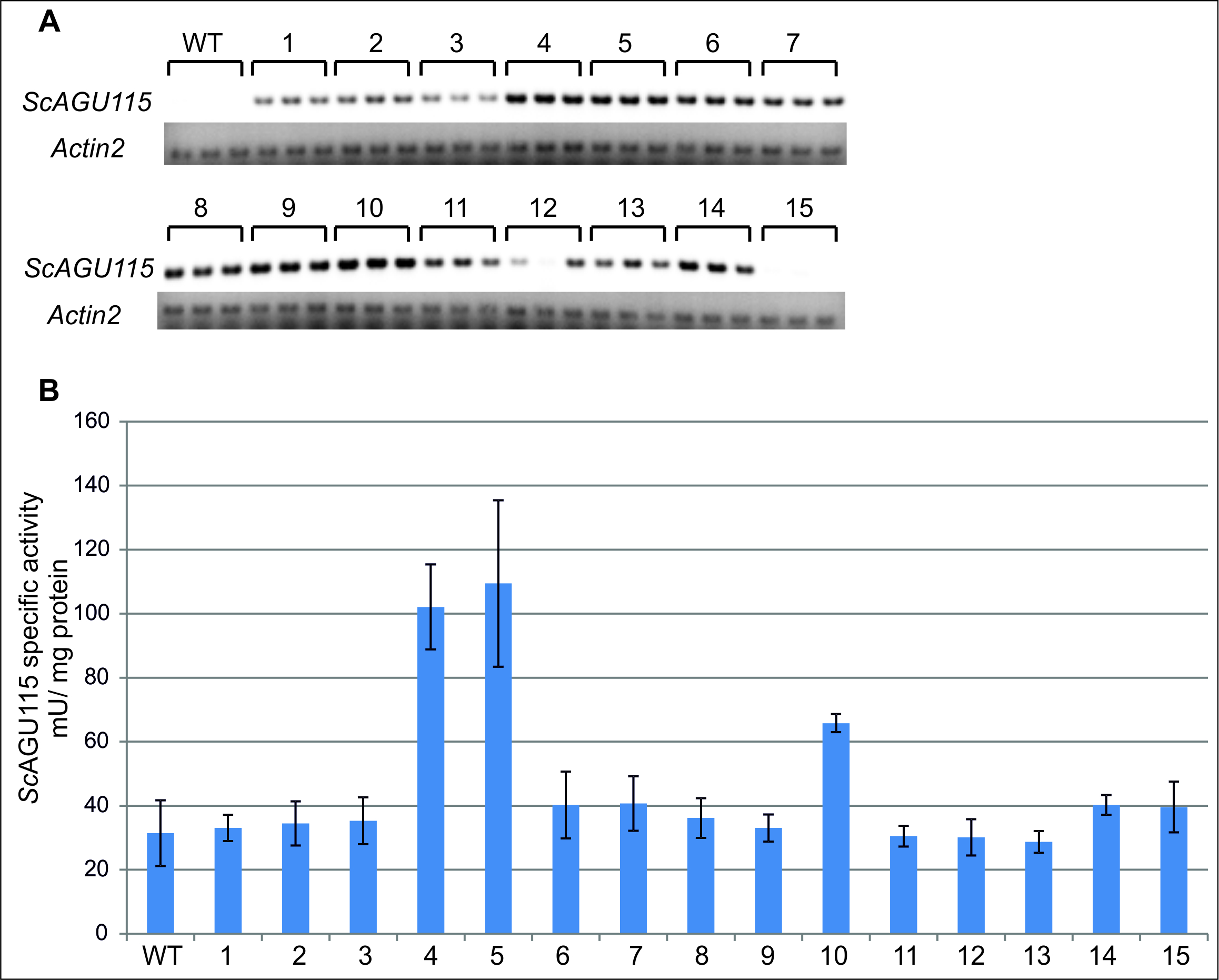

Supplement: Additional file 2: Figure S2. — Screening of Arabidopsis homozygotic lines expressing ScAGU115 α-glucuronidase. (A) Gene expression analysis and amplification of Actin2 gene was used as the internal control. (B) Soluble protein was extracted from stem tissues of the 7 weeks old plants, and was analyzed for the α-glucuronidase activity using Megazyme kit. The error bar represents the standard deviation of three biological replicates. [file 12896_2015_154_MOESM2_ESM.jpeg]

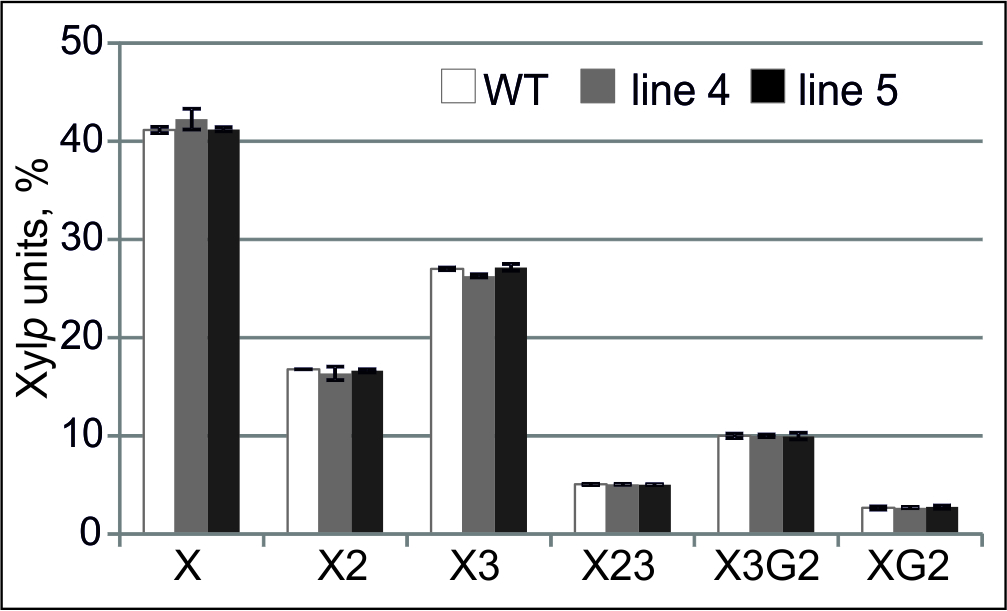

Supplement: Additional file 3: Figure S3. — Relative content of substituted and non-substituted internal Xylp residues in the O-acetylglucuronoxylan of transgenic (lines 4, 5) and WT plants based on integration of cross peaks in 2D qHSQC spectra. X2, [2-O-Ac]-β-D-Xylp; X3, [3-O-Ac]-β-D-Xylp; X23, [2,3-O-Ac]-β-D-Xylp; X3G2, [α-D-(Me)GlcpA*(1 → 2)][3-O-Ac]-β-D-Xylp; XG2, [α-D-(Me)GlcpA*(1 → 2)]-β-D-Xylp; * representing both GlcpA and MeGlcpA since their cross peak signals were unresolved in the spectra. The error bar represents the standard deviation of two biological replicates. [file 12896_2015_154_MOESM3_ESM.jpeg]

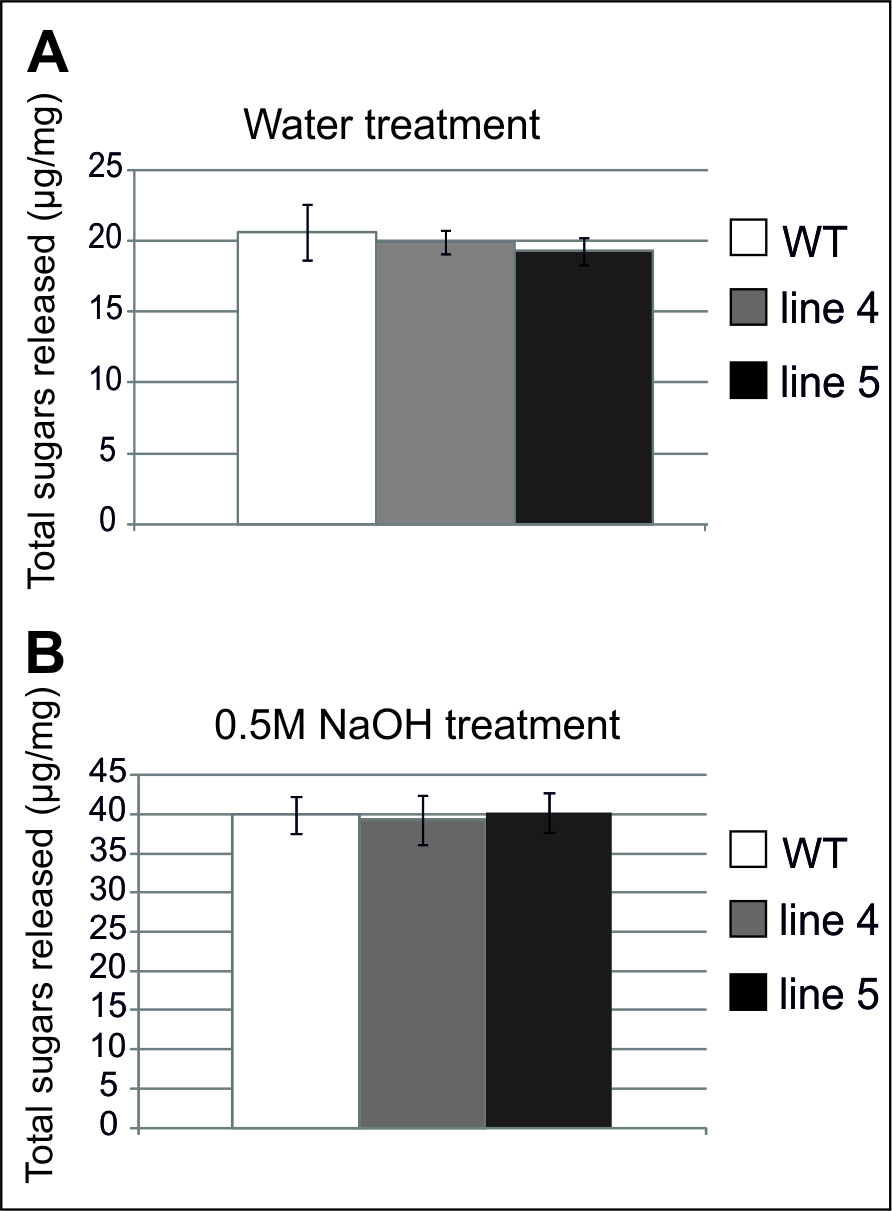

Supplement: Additional file 5: Figure S4. — Digestibility of the main stems from transgenic (lines 4, 5) and WT plants. The main stem tissues were ground and pre-treated by (A) water, and (B) 0.5M NaOH before the enzymatic hydrolysis. Error bar represents standard deviation of three biological replicates. [file 12896_2015_154_MOESM5_ESM.jpeg]
