# Supplementary material for: Active fungal GH115 α-glucuronidase produced in Arabidopsis thaliana affects only the UX1-reactive glucuronate decorations on native glucuronoxylans
Source: BMC Biotechnol. 2015 Jun 18;15:56. doi: 10.1186/s12896-015-0154-8 (PMC4472178; doi:10.1186/s12896-015-0154-8)
Supplement: Additional file 4: Table S1. — Comparison of carbohydrate and lignin components in the stem tissues. The guaiacyl, syringyl and p-hydroxyphenyl lignins; and carbohydrate fraction in the transgenic (lines 4, 5) and WT plants were analyzed by pyrolysis-gas chromatography/ mass spectrometry. The ± represents the standard deviation of five biological replicates. *p value < 0.05, t-test. [file 12896_2015_154_MOESM4_ESM.docx]

**Table S1. Comparison of carbohydrate and lignin components in the stem tissues.** The guaiacyl, syringyl and *p*-hydroxyphenyl lignins; and carbohydrate fraction in the transgenic (line 4 and 5) and WT plants were analyzed by pyrolysis-gas chromatography/ mass spectrometry. The ± represents standard deviation of five biological replicates. **p* value < 0.05, t-test

| **Genotype** | **Carbohydrate** | **Guaiacyl** | **Syringyl** | ***p*-Hydroxyphenyl** |
| --- | --- | --- | --- | --- |
| **WT** | 81.4 ± 0.7 | 10.5 ± 0.5 | 2.9 ± 0.2 | 1.36 ± 0.04 |
| **line 4** | 81.4 ± 0.6 | 10.3 ± 0.5 | 2.9 ± 0.2 | 1.41 ± 0.04* |
| **line 5** | 81.4 ± 0.9 | 10.1 ± 0.5 | 2.8 ± 0.2 | 1.39 ± 0.05 |
